# Acceptance of AI-based assistive technologies for autonomy support among older adults: a scoping review

**DOI:** 10.1093/gerona/glag238

**Published:** 2026-09-21

**Authors:** Sekou Oumarou Thera, Ali Ben Charif, Jeanne Bertona, Patrick Villani, Sébastien Lechevalier, Marc-Karim Bendiane, Anne-Laure Couderc

**Affiliations:** Department of Internal Medicine, Geriatrics and Therapeutics, Marseille University Hospital (AP-HM), Marseille, France; CNRS, EFS, ADES, Aix Marseille University, France; CubecXpert, Quebec City, Quebec, Canada; Department of Internal Medicine, Geriatrics and Therapeutics, Marseille University Hospital (AP-HM), Marseille, France; Department of Internal Medicine, Geriatrics and Therapeutics, Marseille University Hospital (AP-HM), Marseille, France; CNRS, EFS, ADES, Aix Marseille University, France; Ecole des Hautes Etudies en Sciences Sociales (EHESS), Paris, France & German Institute for Japanese Studies (DIJ), Tokyo, Japan; Department of Internal Medicine, Geriatrics and Therapeutics, Marseille University Hospital (AP-HM), Marseille, France; CNRS, EFS, ADES, Aix Marseille University, France; Department of Internal Medicine, Geriatrics and Therapeutics, Marseille University Hospital (AP-HM), Marseille, France; CNRS, EFS, ADES, Aix Marseille University, France

**Keywords:** Artificial intelligence, Robotics, Successful Aging, Technology acceptance

## Abstract

**Background:**

Population aging is leading to increased demand for health and long-term care. Artificial intelligence (AI)-based assistive technologies have been developed to support autonomy and care for older adults, yet real‑world adoption remains limited. We review evidence on acceptance and determinants of acceptance of AI‑based assistive technologies among older adults.

**Methods:**

We conducted a scoping review using the Joanna Briggs Institute methodology and followed Preferred Reporting Items for Systematic reviews and Meta-Analyses‑Scoping Review guidelines for reporting. Four databases (PubMed, Embase, Web of Science, Cochrane) were searched for studies published from January 2010 to June 2025, initially in any language. We included studies examining acceptance and determinants of acceptance of AI-based assistive technologies among adults aged 65 years and older. We synthesized data using inductive thematic analysis.

**Results:**

Twenty‑nine studies met the inclusion criteria, with over one-third conducted in Europe (*n* = 10). Overall attitudes toward AI‑based assistive technologies were generally favorable when technologies were perceived as useful, easy to use, and tailored to needs. However, actual use was low. Acceptance was associated with socio‑demographic (eg, age, residence), medical (eg, frailty, self‑rated health), psychosocial (eg, stigma), and structural (eg, cost, accessibility) determinants, as well as with institutional and relational factors (eg, preservation of human contact).

**Conclusions:**

While older adults have generally positive views of AI‑based assistive technologies, adoption is slow because of intersecting psychosocial, economic, technical, and organizational barriers. User‑centered design, care-led innovation approaches, and implementation strategies that complement—rather than replace—human support may facilitate uptake and sustained use. Development of standardized assessment tools is needed to enable cross-study comparability and guide future research.

## Introduction

Population aging and increased life expectancy are major emerging challenges worldwide, but especially in high-income countries. By 2050, 1 in 6 people worldwide will be 65 or older. The global population of over-65s is projected to rise from 703 million in 2019 to 1.5 billion in 2050.^1^ This demographic shift is already placing an unprecedented strain on health and social care systems, primarily due to the long-term care needs of older adults.^2^ Chronic diseases such as diabetes, chronic respiratory diseases, nervous system disorders, and cardiovascular diseases are also more prevalent in older adults.^3^ In the United States, 23.9% of over-65s have a chronic disease.^4^ The corresponding rates in Europe are 28.2% for men and 34.5% for women,^5^ and are even higher in Northern European countries (eg, 53.7% in Germany).^6^ All countries face major challenges in adapting their health and social care systems to effectively respond to this demographic transition. Public health policies are therefore necessary to meet the heterogeneous needs and preserve the autonomy and well-being of increasing numbers of older adults.^7^

Artificial intelligence is an umbrella term for a range of computational approaches designed to learn from data and simulate human cognitive functions, notably through machine learning, deep learning, and, more recently, generative AI.^8^ In the context of aging and assistive technologies, AI systems have the potential to perceive the environment, process data, and adapt the responses to support users’ need.^9^^,^^10^

This review focuses on AI tools for health and autonomy support (known AI‑based assistive technologies), including socially assistive robots, smart monitoring systems, intelligent mobility aids, voice assistants, and predictive applications.^9^^,^^11^ Artificial intelligence‑based assistive technologies are increasingly being developed to help older adults with activities of daily living (ADL), perform health monitoring, and enhance overall well-being.^12–14^ However, despite the perceived benefits of AI‑based assistive technologies, acceptance and real-world use by older adults remain limited.^15^^,^^16^ Studies in the social and human sciences have highlighted that underuse of AI and robotics often reflects unfamiliarity with technological innovations, the gap between technological designs and users’ actual needs, and developers’ lack of consideration of cultural and social specificities.^17^ Economic, social, and psychological factors have been associated with the acceptance of AI and robotic technologies among older adults.^18^

Given the rapid growth of AI systems and their potential benefits for older adult autonomy, mapping and synthesizing current data on the acceptance of AI‑based assistive technologies and associated factors (including sociodemographic, medical, technical, economic, and psychological) is essential. Previous reviews have focused on a narrow set of technologies,^15^^,^^19^ or on barriers or facilitators in isolation,^20^ or have included only English-language articles.^2^

This review aims to map the acceptance of AI-based assistive technologies among older adults and to identify determinants (facilitators and barriers) influencing adoption and use. We use “acceptance” to denote willingness to adopt and use the technology,^21^ and we consider adoption and actual use where reported.

## Materials and methods

### Design

We conducted a scoping review following the Joanna Briggs Institute (JBI)^22^ methodology and Preferred Reporting Items for Systematic reviews and Meta-Analyses (PRISMA) Extension for Scoping Reviews guidelines.^23^ This scoping review was conducted as part of a series of systematic reviews whose protocol and methodological components were registered in the International Prospective Register of Systematic Reviews on May 5, 2025 (registration identifier: CRD420251042779).^24^

### Eligibility criteria

Following the JBI framework, eligibility criteria were structured according to the Population, Concept, Context, and Type (PCCT) of sources framework, as follows.

- Population (P): Studies involving adults aged 65 years and older, regardless of their country of residence, health status, or lifestyle, were included. The threshold of 65 years was chosen because it corresponds to the age at which loss of autonomy and chronic disabling conditions become most prevalent in high-income countries.^25^^,^^26^ It is also the standard threshold used by geriatric specialists and major statistical agencies, frailty research, and clinical trials involving older adults.^27–29^ Studies involving participants aged under 65 years were excluded.
- Concept (C): Studies focusing on acceptance of AI‑based assistive technologies among older adults and associated determinants (facilitators and barriers) were included. Acceptance was defined as the willingness to adopt and use a technology,^21^ encompassing both intended use and actual use where reported. Studies were excluded if they (1) did not assess acceptance or determinants of acceptance of AI-based assistive technologies, or (2) focused on general digital technologies that are not AI-based, such as smartphones, computers, or tablets. Generative AI systems used in contexts other than assistive health, and AI-based in-vehicle services not primarily designed for health or autonomy support, were also excluded.
- Context (C): Studies focusing on autonomy support of older adults, regardless of the care setting (eg, nursing homes, hospitals, or community settings). Autonomy was operationalized following the World Health Organization International Classification of Functioning, Disability and Health, which defines loss of autonomy as a process through which diseases alter physical, sensory, or cognitive functions, generating restrictions in activities.^30^ Autonomy support was thus defined as the capacity of AI-based assistive technologies to compensate for or prevent restrictions in ADL^31^ and/or Instrumental activities of daily living (IADL).^32^ Studies were included when the technology under investigation explicitly targeted one or more of these dimensions of autonomy. Studies in which the technology did not primarily aim at autonomy support were excluded.
- Types of sources (T): Only primary empirical studies (qualitative, quantitative, or mixed) published from January 1, 2010 were eligible for inclusion. The starting date was selected because it corresponds to a recognized turning point in the gerontechnology literature, marked by substantial advances in AI and machine learning that enabled the development of intelligent assistive systems.^11^ The following publication types were excluded: editorials, opinion pieces, commentaries, and protocols without primary data, narrative reviews, scoping reviews, systematic reviews, and meta-analyses. Studies that focused on statistical methods and did not collect participants’ views or experiences were also excluded. Only studies published in English or French were included.

### Research strategy

In collaboration with a health sciences librarian, a comprehensive search strategy was developed to ensure optimal coverage of relevant studies. The search was conducted in 4 electronic databases: PubMed, Cochrane Library, Embase, and Web of Science. The search strategy combined both controlled vocabulary (MeSH terms) and free-text keywords, focusing on 4 main areas: (1) Older adults, (2) AI-based assistive technology, (3) autonomy support, and (4) acceptance and determinants (facilitators and barriers). First, the search strategy was developed for PubMed, then adapted for use in Cochrane and the other selected databases. The search strategy used for PubMed is described in full in Appendix S1.

To collect the most relevant data on the development and implementation of AI-based assistive technologies in aging populations, we limited the search to studies published between January 1, 2010, and June 30, 2025. No language restrictions were applied during the initial search phase. In addition, manual searches of the reference lists of included articles were conducted to identify any additional relevant studies potentially missed by the developed search strategy.

### Selection of evidence sources

We created a study selection form in Google Sheets and operationalized eligibility criteria using questions with the following responses: “yes” (include), “no” (exclude), or “uncertain.” All records identified during the searches were imported and managed in Zotero software (https://www.zotero.org; Corporation for Digital Scholarship, Fairfax, VA, USA). Duplicates were removed prior to screening. The selection process was conducted in 2 stages. First, a single reviewer (S.O.T.) screened all records based on titles and abstracts against the eligibility criteria. A second reviewer (J.B., A.-L.C., or M.-K.B.) then verified all these assessments. Second, all records retained after title and abstract screening were retrieved in full text. A single reviewer (S.O.T.) assessed all full-text reports against the same eligibility criteria, and a second author (J.B., A.-L.C., or M.-K.B.) verified all decisions. Any disagreements were resolved by discussion and consensus between the reviewer (S.O.T.) and the checkers (J.B., A.-L.C., or M.-K.B.). For all excluded full texts, the reasons for exclusion were documented according to PCCT framework and are reported in Appendix S2.

### Data entry process

A data extraction form was prepared in Excel and pilot-tested to ensure that it captured all relevant information. One reviewer (S.O.T.) extracted data from each included report using the Excel form, and the senior author (M.-K.B.) checked the data for accuracy. The following data were extracted: bibliographic information (authors, year, journal, country), study design (qualitative, quantitative, mixed-methods), population characteristics (sample size, mean age), type of AI-based assistive technology, dimensions measured (acceptance and intention to use, perceived usefulness, perceived ease of use, etc.), and measurement tools used.

### Data analysis

The extracted data were analyzed using a narrative synthesis approach, involving inductive content analysis, which is consistent with JBI methodological guidance for scoping reviews.^22^

Study characteristics were gathered in a summary table including bibliographic information, study design, population characteristics, type of AI-based assistive technology, dimensions measured, and measurement tools used.

Themes were identified inductively from the extracted data by 1 reviewer (S.O.T.), with iterative input from a second reviewer (M.-K.B.). Three main thematic areas emerged: (1) acceptance, perceived needs, and actual use; (2) facilitators of acceptance, including sociodemographic, medical, and functional factors; and (3) barriers to acceptance, including psychosocial, economic, technical, institutional, and relational factors.

Results are presented in narrative form, supported by a summary table of included studies. The PRISMA 2020 flowchart was used to describe the record selection process.^33^

### Ethical considerations

Ethical approval was not required as primary data were not collected.

## Results

### Study selection

Our electronic search yielded 8093 potentially relevant records. Of these, 1007 were duplicates, leaving 7086 records for title and abstract screening. Disagreements occurred for 34 records (0.48%) and were resolved by discussion and consensus. Subsequent review of titles and abstracts found that 6957 records did not meet the eligibility criteria. Full-text eligibility review was therefore conducted on the remaining 129 reports. Disagreements occurred for 12 of the 129 reviewed full-text reports (9.3%) and were resolved by discussion and consensus. Twenty-seven of the 129 articles met the inclusion criteria and 2 further articles^34^^,^^35^ were identified through manual searches of reference lists. Overall therefore, 29 articles^34–62^ were included in the review (Figure 1). The others articles were excluded because they involved participants younger than 65 years, did not assess acceptance or determinants, focused on general digital technologies not based on AI, did not target autonomy support of older adults, or were not primary empirical studies (Figure 1).

**Figure 1:**
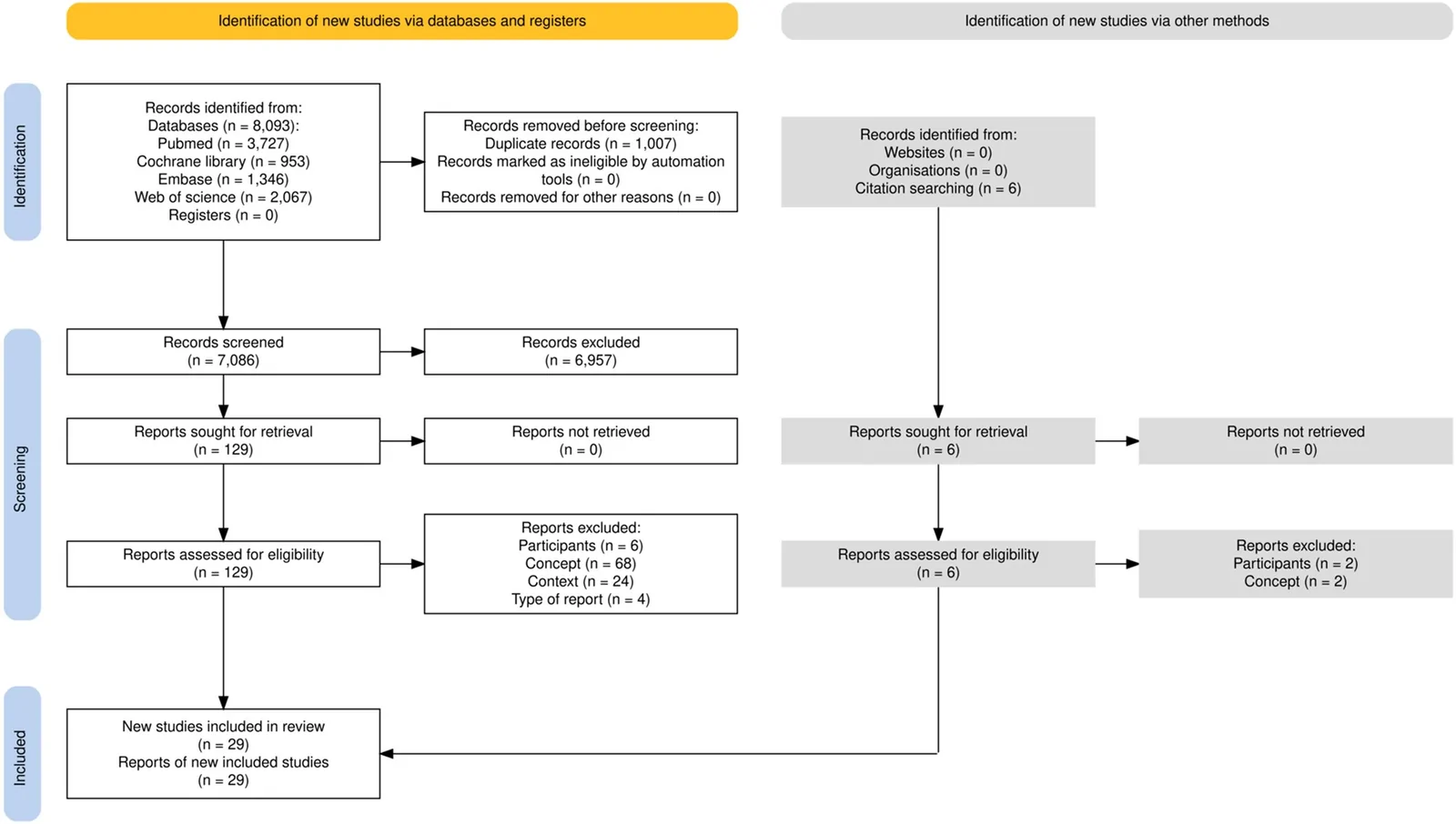
PRISMA 2020 Flow diagram of the selection process.

### General characteristics of included studies

The 29 included studies varied in study design, type of AI-based assistive technology examined, measured dimensions, and analytical approaches. Twelve of the included studies were qualitative,^34^^,^^35^^,^^41^^,^^43^^,^^44^^,^^46^^,^^47^^,^^52^^,^^54^^,^^57^^,^^59^^,^^62^ 11 were quantitative,^36–38^^,^^42^^,^^45^^,^^48^^,^^49^^,^^51^^,^^55^^,^^56^^,^^60^ and 6 followed a mixed approach.^39^^,^^40^^,^^50^^,^^53^^,^^58^^,^^61^ Ten of the studies were conducted in Europe (France, Belgium, Italy),^34^^,^^38^^,^^40^^,^^41^^,^^45^^,^^49^^,^^51^^,^^52^^,^^58^^,^^62^ 9 in Asia (Japan, Korea, China),^36^^,^^37^^,^^39^^,^^44^^,^^50^^,^^55^^,^^57^^,^^59^^,^^61^ 7 in North America (Canada, United States),^43^^,^^46–48^^,^^53^^,^^56^^,^^60^ 1 in New Zealand,^54^ and 2 were multicentric.^35^^,^^42^

The included studies examined 5 main categories of AI-based assistive technologies: (1) social and assistive robots, including home-care robots, humanoid robots, and companion robots (*n* = 17)^34–36^^,^^38–41^^,^^45^^,^^48^^,^^50^^,^^52^^,^^54^^,^^58–62^; (2) general assistive technologies and devices, including assistive applications and assistive technology devices (*n* = 6)^37^^,^^42^^,^^43^^,^^47^^,^^56^^,^^57^; (3) connected health and smart monitoring technologies, including AI-based wearables and intelligent sensor systems (*n* = 3)^44^^,^^51^^,^^55^; (4) voice-activated personal assistants (*n* = 1)^46^; and (5) technology-enabled mobility devices (*n* = 1)^53^. One study examined an assistance system that spanned multiple categories (*n* = 1)^49^. Social and assistive robots, investigated in more than half of the included studies, were the most frequently studied technology category (Table 1).

**Table 1:** Characteristics of included studies.

Author, year, [reference]	Journal	Country	Study design	Population (*n*, mean age)	AI technology studied	Measured dimension	Measuring tools
Shin et al. (2023)^36^	*J Med Internet* *Res*	South Korea	Quantitative	500 older adults, 66.87 ± 8.72 years	Daily living assistive technologies	Use and acceptance	Questionnaire designed by the authors
Yu et al. (2022)^37^	*RSPH Public Health*	China	Quantitative	5790 older adults, NA	Assistive devices	Use, self-perceived need and attitudes	Questionnaire designed by the authors
Kodate et al. (2022)^38^	*Health Soc Care Community*	Ireland	Quantitative	178 older adults, NA	Home-care robots	Views and perceptions	Questionnaire designed by the authors
Park et al. (2019)^39^	*BMC Geriatr*	Korea	Mixed-method	234 older adults, 75.7 ± 5.8 years	Robot technology	Acceptance, needs, and perception	Questionnaire and guide designed by the authors
Vagnetti et al. (2024)^40^	*JMIR Aging*	United Kingdom	Mixed-method	31 older adults, HG: 10, 75.5 years; MDG: 10, 71.5 years; LSG: 11, 69.0 years	SARs and monitoring technologies	Attitudes	MdRAS
Wu et al. (2014)^58^	*Clin Interv Aging*	France	Mixed-method	11 older adults, 79.3 years	Kompaï robot	Robot acceptance	Questionnaire developed by the authors, based on an adapted model of the unified theory of acceptance and use of technology.
Wilkowska et al. (2021)^42^	*Front Public Health*	Sweden, Germany, Spain, Italy, and Canada	Quantitative	384 older adults, 67.6 ± 5.5 years	Assistive applications and functions	Acceptance and intention to use	Online questionnaire designed by the authors
Orellano-Colón et al. (2024)^47^	*Disabil Rehabil Assist Technol*	Puerto Rico	Qualitative	24 older adults, 73 years	AT	Factors influencing AT use	Interview guide developed by the authors
Wong et al. (2025)^44^	*JMIR Aging*	China	Qualitative	27 older adults, 69.44 ± 6.7 years	AI-based health technologies (Google Fitbit, Apple Watch, etc.)	Perceptions and attitudes	Interview guide developed by the authors
Tobis et al. (2023)^45^	*J Med Internet Res*	Poland	Quantitative	48 older adults, (75.5 ± 8.7 years)	Humanoid social robot	The ease of use of technological devices	UNRAQ and GQS
Orlofsky and Wozniak (2022)^46^	*Geriatr Nurs*	United States	Qualitative	12 older adults, 76.83 years	Voice-activated personal assistants (Amazon’s Alexa)	Acquisition, adoption, use, and perceptions	Interview guide developed by the authors
Orellano-Colón et al. (2016)^43^	*J Racial Ethn Health Disparities*	Puerto Rico	Qualitative	60 older adults, 77.4 years	ATD	Barriers to using ATD	Interview guide developed by the authors
Beer et al. (2017)^48^	*Gerontechnology*	United States	Quantitative	12 older adults, 72.58 ± 3.87 years	PR2	Participants’ attitudinal acceptance	Questionnaire designed by the authors
Perotti and Strutz (2023)^49^	*Z Gerontol Geriatr*	Berlin	Quantitative	106 older adults, 73.78 ± 5.138 years	AuRorA assistance system	Accessibility, intention to use	TUI
Zhao et al. (2025)^50^	*Innov Aging*	China	Mixed-method	30 older adults, NA	Humanoid social robot (KaKa robot)	General acceptability	Questionnaire and guide designed by the authors, informed by the TFA.
Cohen et al. (2016)^51^	*Patient Prefer Adherence*	Switzerland	Quantitative	34 older adults, 83.2 ± 7.2	IWSS	Acceptability and the intentions to use	Questionnaire designed by the authors
Li et al. (2022)^55^	*Front Psychiatry*	China	Quantitative	311 older adults, 70.15 ± 7.13 years	Smart health technologies	Attitudes, perceptions, and behavioral intentions	Structured questionnaire and empirical analysis developed by the authors
Bluethmann et al. (2023)^53^	*J Geriatr Oncol*	United States	Mixed-method	12 older adults, 74 years	Technology-enabled mobility devices (eg, the smart cane)	Perceptions related to acceptability, usability	Questionnaire and guide designed by the authors
Fiorini et al. (2019)^34^	*Disabil Rehabil Assist Technol*	Italy	Qualitative	20 older adults, 78.1 ± 7.9 years	Assistive robot (ASTRO robot)	Needs and attitudes	Guide designed by the authors
Gasteiger et al. (2021)^54^	*Assistive Technology*	New Zealand	Qualitative	6 older adults, 78 years	The robot, Bomy (version 2)	The use and perceptions of usefulness	Guide designed by the authors
Li et al. (2023)^52^	*Scientific Reports*	United Kingdom	Qualitative	17 older adults, 75.29 ± 6.85 years	Human-following robot	Needs and requirements	Guide designed by the authors
Vitlin-Stein et al. (2024)^56^	*Disability and Rehabilitation: Assistive Technology*	United States	Quantitative	102 older adults, 73 ± 6.91	AT	Need for AP, access to it and satisfaction with it	Online survey created by the authors, using 33 questions from the WHO’s rATA
Goodarzi et al. (2024)^57^	*Disability and Rehabilitation: Assistive Technology*	Iran	Qualitative	30 older adults, NA	Assistive technologies	Social influence, Facilitating conditions, Perceived need, Behavioral intention	Guide designed by the authors
Wu et al. (2016)^41^	*J Appl Gerontol*	France	Qualitative	20 older adults (5 for FGD, 15 for IDI), 73 years, 76.3 years	Assistive robot	Images, attitudes, and opinions	Guide designed by the authors
Huang and Huang (2021)^59^	*Work*	China	Qualitative	36 older adults, 72 ± 6.10 years	Buddy Robot	Perceived ease of use, perceived usefulness, and attitude	Robot assistance attitude questionnaire and robot assistance acceptance questionnaire of ADLs
Louie et al. (2014)^60^	*Assist Technol*	Canada	Quantitative	66 older adults, 76 ± 7.2 years	The human-like socially assistive robot (Brian 2.1)	Acceptance and attitudes	Robot acceptance questionnaire
Chu et al. (2019)^61^	*Gerontology*	China	Mixed-method	33 older adults, 66.3 years	Robots	Robot acceptance	UTAUT questionnaire
Van Assche et al. (2024)^62^	*Disabil Rehabil Assist Technol*	Belgium	Qualitative	30 older adults, 82 years	SARs	Attitude, Perceived Barriers, Perceived Facilitators	Guide designed by the authors
D’Onofrio et al. (2019)^35^	*Aging Clin Exp Res*	Italy and Japan	Qualitative	10 older adults, 73.9 ± 5.6 years	BUDDY robot (assistive robot)	Socialization needs and perception of robots	Guide designed by the authors

Abbreviations: AT, assistive technology; ATD, assistive technology devices; GQS, Godspeed Questionnaire Series; IWSS, Intelligent Wireless Sensor System; MdRAS, Multidimensional Robot Attitude Scale; PR2, Personal Robot 2; SARs, social assistive robots; TUI, Technology Usage Inventory; UNRAQ, Users’ Needs, Requirements and Abilities Questionnaire.

The dimensions measured were mainly acceptability, intention to use, perceived usefulness, perceived ease of use, and attitude toward these technologies. In most studies, the tools used to measure these dimensions were questionnaires and/or interview guides developed by the authors. Only a few studies used standardized questionnaires, notably the Users’ Needs, Requirements and Abilities Questionnaire and Godspeed Questionnaire Series used by Tobis et al.,^45^ the Technology Usage Inventory used by Perotti et al.,^49^ and the UTAUT questionnaire used by Chu et al.^61^ (Table 1).

All studies focused exclusively on over-65s, but the reporting of age varied between studies. Some studies reported mean age,^34–36^^,^^39^^,^^41^^,^^42^^,^^44^^,^^45^^,^^48^^,^^49^^,^^51^^,^^52^^,^^55^^,^^56^^,^^59^^,^^60^ others reported age ranges,^40^^,^^41^^,^^43^^,^^47^^,^^53^^,^^54^^,^^58^^,^^61^^,^^62^ and others did not specify participants’ age^37^^,^^38^^,^^50^^,^^57^ (Table 1).

The number of studies included per year of publication is shown in Figure 2. The studies were published between 2014 and 2025, with a highest frequency of publications in 2023 (*n* = 5). Data for 2025 cover the period from January 1 to June 30 only (the inclusion period ended on June 30, 2025; Figure 2).

**Figure 2:**
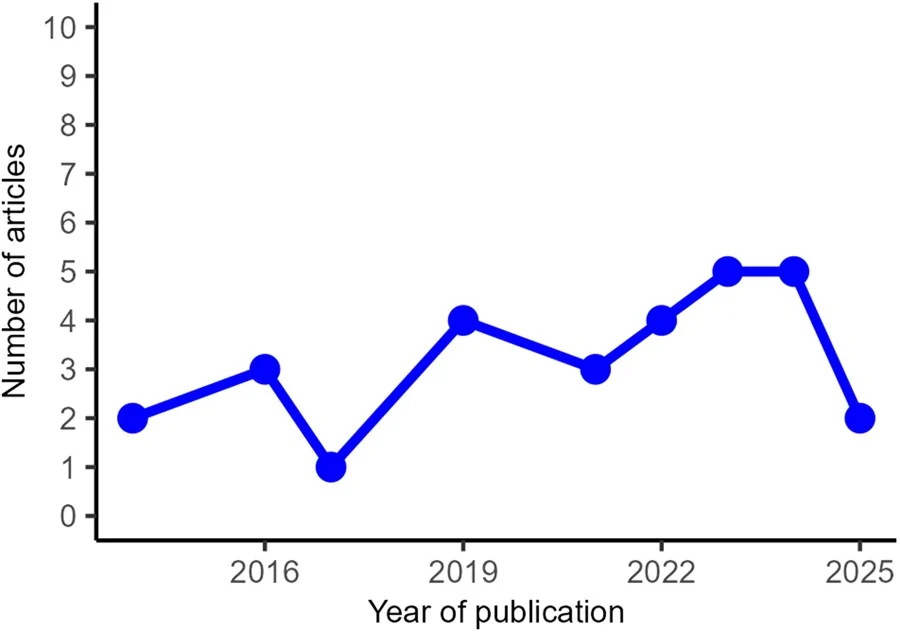
Number of studies included based on year of publication.

### Measures of acceptance, needs, and actual use

#### Acceptance and reservations

In general, assistive technologies were well accepted by participants, especially when their features were perceived as well suited to users’ needs and abilities.^36^^,^^39–41^^,^^45^^,^^46^^,^^48^^,^^54^^,^^57–60^ However, several studies found that some older adults remained skeptical about the technologies’ usefulness to them personally. These adults considered these technologies to be beneficial for other groups, such as the very elderly, isolated individuals, or those with significant functional limitations. However, they did not consider these technologies to be beneficial for themselves because they considered themselves to be independent, healthy, and relatively mobile.^34^^,^^41^^,^^52^ Acceptance that is conditional, or viewing technologies as useful for others but not for oneself, is a recurring pattern across the included studies and is closely related to the psychosocial barriers discussed in"the Psychosocial factors section.”

#### Perceived needs

Several participants indicated that AI-based assistive technologies could become relevant if their situation changed in the future, particularly as they grew older or lost autonomy.^41^^,^^52^^,^^59^ This perception of potential future use was recurrent in studies examining the acceptance of assistive robots. One such study, by Beer et al.’s,^48^ involved 12 older adults (mean age 72.6 ± 3.9 years) and found that participants were open to using robots in the event of future functional decline. However, a lack of enthusiasm for certain proposed features was also reported. In Wu et al.’s,^41^ study of 20 older adults in France, some participants felt that the services offered by assistive robots were already provided by existing technologies such as mobile phones, computers, electronic calendars, and global positioning systems devices, and did not perceive any specific added value compared to these existing tools.

#### Actual and intended use

Despite favorable acceptance and moderately expressed needs, the results indicate that the actual use of these technologies remains low overall. In a quantitative study by Yu et al.’s ^37^ of 5790 older adults in China, the prevalence of assistive device use among participants was only 10.9% (*n* = 634), while more than half of the respondents (53.9%, *n* = 3120) reported no need for such devices. In another quantitative study, Kodate et al.^38^ found that, among 178 older adults in Ireland, that 80.9% (*n* = 144) had never used care robots. Nevertheless, 70.2% of respondents (*n* = 125) reported being open to using care robots at home.

Intention to use these devices appears to be strongly influenced by respondents’ perceived autonomy. In a mixed methods study of 11 older adults in France (mean age 79.3 years), Wu et al.^58^ found that none of the participants envisioned using an assistive robot in near future because they considered themselves autonomous. In the same study, however, varying levels of future use were reported; some participants considered these technologies a last resort in the event of loss of autonomy.^58^ Other studies have highlighted both curiosity about new assistive technologies^50^ and a desire to acquire knowledge for their appropriate use.^45^ Some participants also felt capable of using an assistive robot, even without prior experience with other digital technologies such as computers, tablets, and smartphones.^34^^,^^35^

### Factors promoting acceptance (facilitators)

The following facilitators were identified in the included studies. These factors help explain the favorable acceptance of AI-based assistive technologies reported in “the Acceptance and reservations section, ” at least in part, and contribute to our understanding of why some older adults express a willingness to use them despite limited actual adoption (see“the Actual and intended use section”).

#### Socio-demographic factors

Three studies highlighted the important role of socio-demographic characteristics in acceptance of assistive technologies among older adults. In a large quantitative study of 5790 older adults in China, Yu et al.^37^ found that education level, age, occupation, financial status, and place of residence were significantly associated with the use of assistive devices in a multivariable analysis (*p* < .05). Some studies have found that acceptance of assistive robots tends to increase with age, particularly when these technologies are perceived as useful and adapted to aging-related needs.^48^^,^^50^ Conversely, other studies reported that some participants considered themselves too old to learn how to use a robot, and identified other target groups, such as younger people or those with more significant functional limitations, as being more likely to benefit from these technologies.^58^^,^^62^ Gender was not identified as a significant factor in any of the included studies. In a quantitative study of 178 older adults in Ireland, Kodate et al.^38^ found that approximately 70% of participants reported being open to using home care robots regardless of gender. Living circumstances also appeared to be an important factor: living with another older adult was associated with more frequent use of home care robots, suggesting that cohabitation could promote social acceptance. In a quantitative study conducted of 311 older adults (mean age 70.2 ± 7.1 years) in China, Li et al.^55^ found high levels of willingness to use assistive technologies among those older adults living in rural areas.

#### Medical and functional factors

Health status and level of functional dependence were associated with acceptance and behavioral intention to use assistive technologies. In a quantitative study of 500 older adults in South Korea (mean age 66.9 ± 8.7 years), Shin et al.^36^ found that the intention to use assistive technologies was associated with self-reported health status and psychological function (life satisfaction). These associations varied depending on the degree of frailty (robust, pre-frail, and frail individuals). Among pre-frail older adults, only self-reported health status was negatively correlated with behavioral intention (*r* = −0.169; *p* = .01). Among robust group, only psychological function (life satisfaction) was negatively associated with behavioral intention.^36^ In a quantitative study of 384 older adults (mean age 67.6 ± 5.5 years) from 5 countries (Sweden, Germany, Spain, Italy, and Canada), Wilkowska et al.^42^ found that individual health status was associated with the actual use of assistive technologies. However, perceptions of these technologies were ambivalent: some participants associated assistive robots with disability, loneliness, and dependence, and use of a companion robot was sometimes perceived as a reminder of current or future frailty.^41^ Consequently, emotional and social support emerged as one of the key motivators for using these technologies. According to the participants’ reports, support from peers and family members reinforced their intention to accept and use these technologies. Presence of friends and family promoted confidence, reduced anxiety about use, and facilitated adoption.^57^

### Factors discouraging acceptance (barriers)

The following barriers were identified in the included studies. These factors help explain the reported gap between favorable acceptance and limited actual use, as discussed in “the Actual and intended use section.” They should be considered alongside the facilitators described in “the Factors promoting acceptance (facilitators) section” when interpreting the overall pattern of findings.

#### Psychosocial factors

Several studies have identified psychosocial factors that prevent older adults from the accepting assistive technologies. These factors included perceived personal limitations, negative attitudes toward technology, and insufficient social support. Individual barriers frequently reported included physical difficulties related to aging, fear of technology, sensory impairments, and cognitive limitations.^40^^,^^51^^,^^53^^,^^57^^,^^62^

Several studies found that acceptance remained conditional despite an overall positive attitude. For instance, in a qualitative study of the ASTRO robot among 20 older adults in Italy (mean age 78.1 ± 7.9 years)^34^ revealed that most participants had a favorable attitude based on the belief that the robot could enhance autonomy, safety, and reduce the burden on formal and informal caregivers. However, owning or having to use a robot was also often perceived as an explicit admission of cognitive or physical problems, both by the respondents and by those around them. The robot was seen as making functional limitations more apparent, thereby exposing older adults to potential social stigma. Some participants expressed the concern that using a robot would further highlight their frailties in the eyes of others.^62^

Three recurring obstacles emerged across studies: (1) negative perceptions of aging and the association the use of assistive technology with dependence, isolation and loneliness. These perceptions that were considered stigmatizing and incompatible with participants’ personal identities^34^^,^^41^; (2) lack of familiarity with technology. Many participants emphasized that their generation has had little exposure to digital tools. The lack of experience and digital confidence makes these tools difficult to use^54^; and (3) ethical and societal concerns, particularly related to surveillance, privacy protection, and continuous monitoring of daily activities, which are perceived as intrusive.^46^

Additional psychosocial barriers were identified in several studies. Despite an overall positive opinion, limited knowledge and understanding of assistive technologies were reported as barriers. In a qualitative study of 12 older adults (mean age 74 years) in the United States, Bluethmann et al.^53^ found that some participants felt that the use of these technologies could damage the self-image and self-confidence among of individuals with disabilities. Others described the initial use as difficult and stressful, but noted that the technologies became more acceptable over time.^57^ Finally, some older adults confused AI-based assistive technologies with more general digital tools, such as smartphones, reflecting their limited familiarity with these devices.^44^

#### Economic and technical factors

Several studies identified economic and technical factors as significant barriers to acceptance and use. In economic terms, affordability appeared to be a key determinant. The high cost of devices, their limited availability, and personal financial constraints were frequently reported as barriers to acceptance and use.^43^^,^^49^^,^^53^^,^^56^ Some participants suggested alternatives, such as renting devices to limit costs^50^ or seeking financial support from family.^62^ Technical constraints were also considered significant barriers. In a quantitative study in Puerto Rico with 24 older adults (mean age 73 years), Orellano-Colón et al.^47^ found that 43% of participants experienced poor performance and discomfort when using the devices, and 13% found them unattractive. Another study highlighted several other technical barriers, including devices complexity, frequent malfunctions, unappealing esthetics, and a mismatch between offered features and actual user needs.^43^ In a mixed methods study in China, Chu et al.^61^ found that 25 of 33 older adults (mean age 66.3 years) expressed concerns: 14 feared they would not be able to handle malfunctions or maintenance of the robot, 10 feared they would not be able to afford the cost of a sophisticated robot and its electricity consumption, and 6 expressed concerns about the potential privacy risks associated with continuous collection of personal data in the home.

#### Institutional and relational barriers

Barriers related to institutional systems were identified, including lack of funding, bureaucratic burdens when acquiring AI devices, and the lack of prescriptions or recommendations for these devices by healthcare professionals.^43^^,^^53^ Several participants feared that using robots further would reduce limited social contact with caregivers by replacing certain forms of human support. Participants emphasized the unique nature of human relationships and felt that robots, devoid of sensitivity, intentionality, and genuine emotions, should neither interfere with human relationships, nor replace in-person visits and physical contact.^41^

## Discussion

In this review, we mapped the available data on the acceptance of AI-based assistive technologies among older adults and identified the multilevel determinants of this acceptance. Results highlight generally favorable attitudes toward AI-based assistive technologies, which are contrasted by relatively low levels of actual use, and multiple factors associated with acceptance, including sociodemographic factors (age, cohabitation status, place of residence); medical and functional factors (level of dependency, frailty); psychosocial factors (perceptions of aging, social support, stigma); economic and technical factors (cost, accessibility, complexity); and institutional and relational factors (role of healthcare professionals).

The most studies assessed acceptance and actual use with questionnaires or interview guides developed by the authors without referencing a theoretical model explicitly. Nevertheless, the dimensions explored remained largely consistent with those proposed by the TAM and UTAUT models.^21^^,^^63^ This methodological heterogeneity underscores the absence of a standardized and validated tool for assessing the acceptance of AI-based assistive technologies in older adults. Developing such a tool, grounded in established theoretical frameworks and validated across diverse older adult populations, should be viewed as a priority in the field.

In general, participants accepted assistive technologies well.^36^^,^^39–41^^,^^45^^,^^46^^,^^48^^,^^54^^,^^57–60^ These results are consistent with those reported elsewhere in the literature: older adults generally have positive attitudes^64^ toward technologies that are perceived as useful and adapted to their needs and likely to support their autonomy.^19^ Other studies have found higher acceptance for particular tasks such as assistance with daily activities and reducing social isolation.^65^ These results suggest that adapting features to the actual needs of older adults is crucial to ensuring maximum acceptance and support adoption of these technologies.

However, expressing acceptance of AI-based assistive technologies does not guarantee actual use, which remains very low.^37^ The majority do not plan to use assistive technologies in the short term because they perceive themselves as sufficiently independent.^58^ This gap between stated acceptance and actual use is a central finding of this review. It reflects the well-documented intention-behavior gap observed in the field of health-related technological innovations.^66^ Several studies highlight that the low adoption of assistive technologies, despite positive attitudes, is mainly due to practical factors, such as devices complexity, lack of digital skills, fear of breakdowns, and/or lack of social support.^18^^,^^67^ Furthermore, this gap could be partly explained by the acceptability-centered approach itself, which links technologies to social needs only after they have been developed. Conversely, the care-centered innovation approach emphasizes the fact that barriers to acceptance are difficult to remove after technologies are designed. Instead, care should be placed at the heart of the design process from the beginning, aligning technological solutions with users’ needs and daily routines. This approach has been successfully implemented in initiatives supporting the care of older adults in France and Japan, demonstrating that integrating care into the innovation process improves relevance and adoption.^16^^,^^68^ They underscore the importance of accompanying technological deployment with training, ongoing support, and adaptation to users’ real-life contexts, as exemplified by the adoption of digital technologies.^69^ Nevertheless, several older adults indicated that AI-based assistive technologies could become relevant if their situation changes in the future, particularly with advancing age or loss of autonomy.^41^^,^^52^^,^^59^

A multitude of factors influence the acceptance and actual use of AI-based assistive technologies have been identified. Socio-demographic factors such as age, cohabitation status, and place of residence have been associated with assistive device use.^37^ Some studies indicate a positive association between age and acceptance,^48^^,^^50^ whereas others report that some participants viewed themselves as too old to adopt such tools.^58^^,^^62^ This discrepancy may be explained by the heterogeneity of populations grouped under the broad category of “older adults”. Those aged 65-74 years and those aged ≥75 years may differ substantially in functional and psychosocial parameters. Significant differences are likely even within age groups due to variability in the aging process, as well as differences in levels of autonomy. Functional age, perceived health status, and psychosocial characteristics may be more relevant indicators than chronological age alone.^70^ Cohabitation status and place of residence are also important factors. Living with another older adult was associated with more frequent use,^38^ and a high level of willingness to use assistive technologies was found among older adults living in rural areas.^55^ These findings are consistent with those of other studies.^71^^,^^72^ Therefore, dissemination policies should account for regional disparities in access to digital resources.

Health status and level of dependence were the main medical and functional factors associated with acceptance and intention to use. The intention to adopt assistive technologies was associated with self-reported health status^36^ and psychological well-being.^42^ These findings suggest that subjective health perception is at least as important a factor as objective clinical status. New tools for assessing acceptance of assistive technology should incorporate key concepts such as level of functional autonomy and psychological well-being. Chronic diseases also appear to play a key role in the acceptance and use of AI-based assistive technologies. Chronic conditions such as diabetes, chronic respiratory diseases, nervous system disorders, and cardiovascular diseases are highly prevalent among older adults^3^ and are a primary driver of autonomy loss and functional decline.^73^ The relationship between chronic disease burden and technology acceptance is complex and bidirectional. Older adults with chronic conditions that restrict ADL and IADL may perceive AI-based assistive technologies as more relevant and useful. However, the symbolic association between assistive technologies and disability, dependence, and frailty may be particularly pronounced among individuals with chronic condition. Therefore, the framing and presentation of AI-based assistive technologies to older adults with chronic conditions requires careful consideration, to position these tools as enablers of autonomy rather than as markers of decline.^74^ Future research is needed to examine how the type, severity, and functional impact of chronic diseases, either independently or jointly, influence acceptance of AI-based assistive technologies among older adults.

Economic and technical factors also emerged from this study as critical determinants. The high cost of these technologies, limited access, and general financial constraints were frequently cited as barriers.^43^^,^^49^^,^^53^^,^^56^ Some participants suggested workarounds such as renting devices to lower costs,^50^ or receiving financial support from governments or family networks.^62^ These findings demonstrate that adoption depends not only on individual or psychosocial factors, but also on structural and systemic ones. As has been demonstrated for several digital health technologies, incorporating these innovations into reimbursement and subsidy frameworks may contribute to more equitable access.^75^

The adoption of AI-based assistive technologies may be hindered by the devices’ reliability, esthetics, and design.^47^ Technologies that are perceived as complex, awkward, or esthetically stigmatizing are likely to be quickly abandoned, even if the initial intentions were favorable. Prioritizing user-centered design that integrates older adults from the development stages to ensure the technologies are suitable for real needs, easy to use, and esthetically acceptable is crucial.^16^

Our study also revealed institutional and relational challenges associated with integrating robotic technologies into care settings. Participants’ concerns about reduced human contact underscore the importance of the relational aspect of care and suggest that the acceptance of AI technologies hinges on their positioning as complementing rather than replacing in-person care.^41^ Implementation strategies should emphasize the complementary nature of technology and human support, presenting AI devices as tools that support, rather than replace, caregivers.

The main limitations of this study are its design and the study selection process, as well as the heterogeneity of the populations studied. As a scoping review, the primary aim was to map existing evidence and in line with JBI guidance,^22^ a formal assessment of methodological quality was not made. A single reviewer conducted study selection and another reviewer verified it, a pragmatic approach consistent with methodological recommendations.^23^ The heterogeneity of populations and technologies limits the comparability of studies. Most studies used on self-reported measures of acceptance or intention to use, which may introduce social desirability bias and fail to accurately reflect long-term use. The chosen age threshold (≥65 years) is consistent with the gerontological literature in high-income countries, but may limit the relevance of the findings in lower-income settings where aging-related vulnerability may emerge earlier. Finally, longitudinal real-world evaluations remain scarce.

Nevertheless, this review provides a structured and multidimensional synthesis of factors influencing the acceptance of AI-based assistive technologies among older adults. Despite the rapid growth of AI-based assistive technologies and their increasing relevance to aging populations, only 29 primary empirical studies have been published over the past 15-years (2010-2025). These studies reflect the state of the evidence as of June 30, 2025. Recent studies published after this date may not be included, so the findings should be interpreted in light of this evolving landscape. This scoping review can guide future research by providing a structured map of acceptance determinants, emphasizing the need for standardized assessment tools and longitudinal designs, and highlighting the importance of including underrepresented populations.

## Conclusion

This scoping review provides a structured and multidimensional synthesis of the current evidence regarding the acceptance of AI-based assistive technologies among older adults. Although older adults generally view these technologies favorably, adoption remains low due to intersecting psychosocial, economic, technical, and institutional barriers. To bridge the gap between technological promise and real-world adoption, it is crucial to involve older adults in the design, evaluation, and deployment of these technologies. Implementation strategies should prioritize user-centered design, care-led innovation and equitable access models. This positions AI-based assistive technologies as complements to—rather than substitutes for—human care and social support. Consequently, future AI-based assistive technologies must adapt to the needs of older adults with chronic diseases, including their specificities, complications and the associated loss of autonomy, ensuring that technological solutions are tailored to the functional and clinical realities of their users. Addressing these gaps is essential to realizing the full potential of AI-based assistive technologies in supporting the autonomy and well-being of older adults worldwide.

## Supplementary Material

glag238_Supplementary_Data

## Data Availability

All data necessary to support the findings of this review are included in the manuscript and its supplementary materials. Please send all requests for additional data or materials to the corresponding author (Sekou Oumarou Thera, MD, MPH, PhD(c), *SekouOumarou.THERA@ap-hm.fr*).
