# Supplementary material for: Acceptance of AI-based assistive technologies for autonomy support among older adults: a scoping review
Source: J Gerontol A Biol Sci Med Sci. 2026 Sep 21;81(11):glag238. doi: 10.1093/gerona/glag238 (PMC13718428; doi:10.1093/gerona/glag238)
Supplement: Supplementary file 1 [file glag238_supplementary_data.zip › Appendix 2.pdf]

Acceptance of AI-Based Assistive Technologies for Autonomy Support Among Older Adults: A Scoping Review

Numero	First Author, Years	Title	Reasons exclusion
1	(Chung et al., 2023)	Community-dwelling older adults' acceptance of smartwatches for health and location tracking	Wrong context
2	(Peek et al., 2016)	Older Adults' Reasons for Using Technology while Aging in Place	Wrong concept
3	(Smarr et al., 2014)	Domestic Robots for Older Adults: Attitudes, Preferences, and Potential	Wrong concept
4	(Pino et al., 2021)	Interest in social and assistive robotics for elderly subjects	Wrong concept
5	(Farina et al., 2019)	Acceptability and feasibility of wearing activity monitors in community-dwelling older adults with dementia	Wrong context
6	(Hall et al., 2019)	Acceptance and perceived usefulness of robots to assist with activities of daily living and healthcare tasks	Wrong population
7	(Kaur & Chen, 2023)	Exploring {AI} Literacy Among Older Adults.	Wrong concept
8	(Del Toro García et al., 2023)	Evaluating the Energy Requirements of Assistive Technologies for Older Adults.	Wrong concept
9	(Hudson et al., 2020)	Robotic Pet Use Among Community-Dwelling Older Adults.	Wrong concept
10	(Arthanat et al., 2019),	Profiles and Predictors of Smart Home Technology Adoption by Older Adults.	Wrong concept
11	(Silvius et al., 2020)	Effects of Technology Use on Ageing in Place: The {iZi} Pilots.	Wrong context
12	(E. Orellano-Colón et al., 2016)	Assistive technology unmet needs of independent living older Hispanics with functional limitations.	Wrong concept
13	(Monden et al., 2024)	Exploring perspectives on assistive technology use: barriers, facilitators, and access.	Wrong population
14	(Pettersson et al., 2015)	Experiences of using powered wheelchair or powered scooter and accessibility in housings.	Wrong concept
15	(Cavallo et al., 2018)	Robotic Services Acceptance in Smart Environments With Older Adults: User Satisfaction and Acceptability Study.	Wrong context
16	(Harris-Gersten et al., 2023)	Usability and Acceptability of Social Robot Pets Among Community-Dwelling Veterans Living With Dementia and Their Caregivers.	Wrong concept
17	(Pripfl et al., 2016)	Social service robots to support independent living : Experiences from a field trial.	Wrong concept
18	(Koceska et al., 2019)	A Telemedicine Robot System for Assisted and Independent Living.	Wrong concept
19	(Fiorini et al., 2024)	Promoting Older Adults' Independent Living with Assistive Technology: Emotional Requirements, Technostress and Formal Caregivers Perspective.	Wrong concept
20	(Bechtold et al., 2021)	Let's Walk It: Mobility and the Perceived Quality of Life in Older Adults.	Wrong concept
21	(K. Chen, 2020)	Why do older people love and hate assistive technology? – an emotional experience perspective.	Wrong concept

22	(Tobis et al., 2022)	Robots in Eldercare: How Does a Real-World Interaction with the Machine Influence the Perceptions of Older People?	Wrong concept
23	(Bedaf et al., 2018)	A multi-perspective evaluation of a service robot for seniors: the voice of different stakeholders.	Wrong population
24	(Wiegel et al., 2024)	Perceived Benefit and Satisfaction With a Tablet Computer and an Emergency Smartwatch by Older Adults and Their Relatives: Prospective Real-World Pilot Study.	Wrong concept
25	(Doering et al., 2015)	Robotic Companions for Older People: A Case Study in the Wild.	Wrong concept
26	(Lin, Moxley, et al., 2025)	Beyond the Digital Divide: Factors Associated With Adoption of Technologies Related to Aging in Place.	Wrong context
27	(Seelye et al., 2012)	Reactions to a remote-controlled video-communication robot in seniors' homes: a pilot study of feasibility and acceptance.	Wrong concept
28	(Eftring & Frennert, 2016)	Designing a social and assistive robot for seniors.	Wrong concept
29	(Schlomann et al., 2020)	Assistive Technology and Mobile {ICT} Usage Among Oldest-Old Cohorts: Comparison of the Oldest-Old in Private Homes and in Long-Term Care Facilities.	Wrong concept
30	(Lei et al., 2024)	Can Socially Assistive Robots Be Accepted by Older People Living Alone in the Community?: Empirical Findings from a Social Work Project in China.	Wrong context
31	(Werner et al., 2020)	Evaluating the sit-to-stand transfer assistance from a smart walker in older adults with motor impairments.	Wrong context
32	(Pilissy et al., 2017)	Towards a situation-and-user-aware multi-modal motorized toilet system to assist older adults with disabilities: A user requirements study.	Wrong concept
33	(Edwards & Mccluskey, 2010)	A survey of adult power wheelchair and scooter users.	Wrong context
34	(Olatunji et al., 2025)	Perspectives of Older Adults on Assistive Robot Support at Home.	Wrong concept
35	(Ha & Park, 2020)	Factors Affecting the Acceptability of Technology in Health Care Among Older Korean Adults with Multiple Chronic Conditions: A Cross-Sectional Study Adopting the Senior Technology Acceptance Model.	Wrong context
36	(Timon et al., 2024)	Developing Independent Living Support for Older Adults Using Internet of Things and {AI}-Based Systems: Co-Design Study.	Wrong concept
37	(F. Corbett et al., 2021)	Virtual Home Assistant Use and Perceptions of Usefulness by Older Adults and Support Person Dyads.	Wrong context
38	(Sathananthan et al., 2025)	Exploring the challenges in manual wheelchair operation for new users.	Wrong context
39	(Kang et al., 2025)	Exploring Smart Health Wearable Adoption Among Singaporean Older Adults Based on Self-Determination Theory: Web-Based Survey Study.	Wrong concept
40	(Randall et al., 2025)	Assistive Technology Uses and Barriers in the Home and Workplace for Adults With Intellectual and Developmental Disabilities.	Wrong concept

41	(Rantanen et al., 2018)	Attitudes towards care robots among Finnish home care personnel - a comparison of two approaches.	Wrong concept
42	(Werner et al., 2022)	Evaluating the task effectiveness and user satisfaction with different operation modes of an assistive bathing robot in older adults.	Wrong concept
43	(Wong et al., 2024)	Exploring the Experiences of Community-Dwelling Older Adults on Using Wearable Monitoring Devices With Regular Support From Community Health Workers, Nurses, and Social Workers: Qualitative Descriptive Study.	Wrong context
44	(Koceski & Koceska, 2016)	Evaluation of an Assistive Telepresence Robot for Elderly Healthcare.	Wrong concept
45	(Kearns et al., 2022)	Views of wheelchair users and caregivers regarding a passive safety monitoring system for electric powered wheelchair operators with cognitive impairment.	Wrong concept
46	(T. L. Chen et al., 2017)	Older adults' acceptance of a robot for partner dance-based exercise.	Wrong type of study
47	(Jung & Shin, 2024)	Factors associated with intention to use care robots among people with physical disabilities.	Wrong population
48	(Hurmuz et al., 2025)	Factors influencing the use of an artificial intelligence-based app ({selfBACK}) for tailored self-management support among adults with neck and/or low back pain.	Wrong context
49	(Domingos et al., 2022)	Usability, Acceptability, and Satisfaction of a Wearable Activity Tracker in Older Adults: Observational Study in a Real-Life Context in Northern Portugal.	Wrong context
50	(Zhang et al., 2024)	What Is the Real-Life Experience of Older Adults on Smart Healthcare Technologies? An Exploratory Interview Study.	Wrong context
51	(Álvarez-Aguado et al., 2025)	Exploring technology use among older adults with intellectual disabilities: barriers, opportunities, and the role of advanced technologies.	Wrong context
52	(S. Wang et al., 2019)	Technology to Support Aging in Place: Older Adults' Perspectives.	Wrong context
53	(Cabrita et al., 2019)	Older Adults' Attitudes Toward Ambulatory Technology to Support Monitoring and Coaching of Healthy Behaviors: Qualitative Study.	Wrong context
54	(Wilkowska et al., 2022)	Analyzing technology acceptance and perception of privacy in ambient assisted living for using sensor-based technologies.	Wrong context
55	(Möller et al., 2024)	Toward Innovation in Healthcare: An Analysis of the Digital Behavior of Older People in Europe and Japan for the Introduction of a Technological Coaching System.	Wrong context
56	(Johnson et al., 2025)	Feasibility and Acceptability of Smartwatches for Use by Nursing Home Residents.	Wrong population
57	(Sadler et al., 2024)	Social Robots for Meaningful Seated Activities: Acceptance \& Use by Older Adults.	Wrong type of study
58	(Tsai et al., 2015)	Getting Grandma Online: Are Tablets the Answer for Increasing Digital Inclusion for Older Adults in the U.S.?	Wrong concept
59	(Mitseva et al., 2012)	Gerontechnology: providing a helping hand when caring for cognitively impaired older adults-intermediate results from a controlled	Wrong concept

		study on the satisfaction and acceptance of informal caregivers.	
60	(Lesauskaitė et al., 2019)	Older Adults-Potential Users of Technologies.	Wrong concept
61	(Jafari et al., 2022)	Human-like tele-health robotics for older adults - A preliminary feasibility trial and vision.	Wrong concept
62	(Fowe & Boot, 2022)	Understanding older adults' attitudes toward mobile and wearable technologies to support health and cognition.	Wrong concept
63	(Dosso et al., 2022)	User perspectives on emotionally aligned social robots for older adults and persons living with dementia.	Wrong concept
64	(Cao et al., 2022)	Decision-making Factors Toward the Adoption of Smart Home Sensors by Older Adults in Singapore: Mixed Methods Study.	Wrong concept
65	(Orellano-Colon et al., 2024)	Empowering Potential of the My Assistive Technology Guide: Exploring Experiences and User Perspectives.	Wrong population
66	(E. M. Orellano-Colón et al., 2018)	Assistive Technology Needs and Measurement of the Psychosocial Impact of Assistive Technologies for Independent Living of Older Hispanics: Lessons Learned.	Wrong concept
67	(D'Onofrio et al., 2018)	Agile Co-Creation for Robots and Aging ({ACCRA}) Project: new technological solutions for older people.	Wrong population
68	(Chiu et al., 2023)	Identifying Preferred Appearance and Functional Requirements of Aged Care Robots Among Older Chinese Immigrants: Cross-Sectional Study.	Wrong concept
69	(Vaportzis et al., 2017)	Older Adults Perceptions of Technology and Barriers to Interacting with Tablet Computers: A Focus Group Study.	Wrong concept
70	(Smarr et al., 2012)	{OLDER} {ADULTS}' {PREFERENCES} {FOR} {AND} {ACCEPTANCE} {OF} {ROBOT} {ASSISTANCE} {FOR} {EVERYDAY} {LIVING} {TASKS}.	Wrong concept
71	(Martin-Hammond et al., 2019)	Exploring Older Adults' Beliefs About the Use of Intelligent Assistants for Consumer Health Information Management: A Participatory Design Study.	Wrong concept
72	(Sánchez et al., 2019)	Older People's Attitudes And Perspectives Of Welfare Technology In Norway.	Wrong concept
73	(Leung, s. d.)	The Presence of Humanoid Robot With Older Adults at Homes	Wrong population
74	Chen, Ke (K. Chen, 2018)	Assistive Technology and Emotions of Older People - Adopting a Positive and Integrated Design Approach	Wrong concept
75	(Murciano-Hueso et al., 2022)	Use and perception of gerontechnology: differences in a group of Spanish older adults	Wrong concept
76	(Verloo et al., 2020)	Perceptions About Technologies That Help Community-Dwelling Older Adults Remain at Home: Qualitative Study	Wrong concept
77	(Gasteiger et al., 2021)	Robot-Delivered Cognitive Stimulation Games for Older Adults: Usability and Acceptability Evaluation	Wrong concept
78	(Kalınkara et al., 2024)	The factors affecting acceptance of gerontechnological products by older Turkish adults	Wrong concept
79	(Harrington et al., 2023)	Perceptions of socially assistive robots: A pilot study exploring older adults' concerns	Wrong concept

80	(Kadylak & Cotten, 2020)	United States older adults' willingness to use emerging technologies	Wrong context
81	(Ipakchian Askari et al., 2018)	Wear It or Fear It Exploration of Drivers \& Barriers in Smartwatch Acceptance by Senior Citizens	Wrong context
82	(Esmaeilzadeh & Maddah, 2024)	Robotic companions and healthy aging: A mixed-methods exploration of older adults' perspectives and insights	Wrong concept
83	(Van Assche et al., 2024)	Socially Assistive Robots in Aged Care: Expectations of Older Adults with {MCI} in Assisted Living Facilities and Their Caregivers	Wrong concept
84	(Santini et al., 2020)	A Digital Coach Promoting Healthy Aging among Older Adults in Transition to Retirement: Results from a Qualitative Study in Italy	Wrong concept
85	(Huang et al., 2022)	The willingness and influencing factors to choose smart senior care among old adults in China	Wrong concept
86	(Bedaf et al., 2016)	Can a Service Robot Which Supports Independent Living of Older People Disobey a Command? The Views of Older People, Informal Carers and Professional Caregivers on the Acceptability of Robots	Wrong concept
87	(Beer et al., 2012)	The Domesticated Robot: Design Guidelines for Assisting Older Adults to Age in Place	Wrong concept
88	(Li & Woolrych, 2021)	Experiences of Older People and Social Inclusion in Relation to Smart "Age-Friendly" Cities: A Case Study of Chongqing, China	Wrong concept
89	(Silva et al., 2022)	Factors associated with the use of assistive technologies in elders in their home environments	Wrong concept
90	(Le Deist & Latouille, 2016)	Acceptability Conditions for Telemonitoring Gerontechnology in the Elderly Optimising the Development and Use of This New Technology	Wrong context
91	(Koceska & Koceski, 2022)	Supporting elderly's independent living with a mobile robot platform	Wrong concept
92	(Kim et al., 2022)	Exploring user perspectives on a robotic arm with brain–machine interface: A qualitative focus group study	Wrong concept
93	(R. H. Wang et al., 2017)	Robots to assist daily activities: views of older adults with Alzheimer's disease and their caregivers	Wrong concept
94	(Pigini Lucia et al., 2011)	Tele-Operated Robots in Elderly Care at Home: A Survey on Needs and Perceptions of Elderly People and Caregivers - {EVERYDAY} {TECHNOLOGY} {FOR} {INDEPENDENCE} {AND} {CARE}	Wrong concept
95	(Draper et al., 2014)	Ethical Dimensions of Human-Robot Interactions in the Care of Older People: Insights from 21 Focus Groups Convened in the {UK}, France and the Netherlands	Wrong concept
96	(Shareef et al., 2021)	A new health care system enabled by machine intelligence: Elderly people's trust or losing self control	Wrong concept
97	(Lin, Boot, et al., 2025)	Characterizing the attitudes, proficiency, and usage patterns for daily technology among older adults with a cognitive impairment	Wrong concept

98	(Y. Wang et al., 2024)	Factors affecting the elderly's behavioral intention toward smart home systems: A cross-sectional study from China's eastern coast	Wrong concept
99	(Zheng et al., 2022)	The use of everyday and assistive technology in the lives of older autistic adults	Wrong concept
100	(Fang et al., 2021)	Intelligent Robotics for Elderly Assistance in Hong Kong	Wrong concept
101	(Wichmann et al., 2021)	Developing a digital assistive tool for dementia patients and their caregivers: A qualitative study on needs, potential functions, practical requirements, user acceptance, and ethical considerations	Wrong context
102	(Korchut et al., 2017)	Challenges for service robots-requirements of elderly adults with cognitive impairments	Wrong concept
103	(Peters et al., 2025)	Acceptance and Usability of a Soft Robotic, Haptic Feedback Seat for Autonomy Level Transitions in Highly Automated Vehicles	Wrong concept
104	(Jaschinski et al., 2021)	Acceptance of Technologies for Aging in Place: A Conceptual Model	Wrong type of study
105	(Bajones et al., 2020)	Results of Field Trials with a Mobile Service Robot for Older Adults in 16 Private Households	Wrong concept
106	(Penteridis et al., 2017)	Robotic and Sensor Technologies for Mobility in Older People	Wrong type of study

REFERENCES

Álvarez-Aguado, I., Vega Córdova, V., Muñoz La Rivera, F., González-Carrasco, F., Roselló-Peñaloza, M., Espinosa Parra, F., Spencer, H., Farhang, M., Campaña Vilo, K., & Aguado, L. Á. (2025). Exploring technology use among older adults with intellectual disabilities : Barriers, opportunities, and the role of advanced technologies. *Disability and Rehabilitation: Assistive Technology*, *20*(7), 2249-2260. https://doi.org/10.1080/17483107.2025.2498566

Arthanat, S., Wilcox, J., & Macuch, M. (2019). Profiles and Predictors of Smart Home Technology Adoption by Older Adults. *OTJR: Occupational Therapy Journal of Research*, *39*(4), 247-256. https://doi.org/10.1177/1539449218813906

Bajones, M., Fischinger, D., Weiss, A., Puente, P. D. L., Wolf, D., Vincze, M., Körtner, T., Weninger, M., Papoutsakis, K., Michel, D., Qammaz, A., Panteleris, P., Foukarakis, M., Adami, I., Ioannidi, D., Leonidis, A., Antona, M., Argyros, A., Mayer, P., … Frennert, S. (2020). Results of Field Trials with a Mobile Service Robot for Older Adults in 16 Private Households. *ACM Transactions on Human-Robot Interaction*, *9*(2), 1-27. https://doi.org/10.1145/3368554

Bechtold, U., Stauder, N., & Fieder, M. (2021). Let's Walk It : Mobility and the Perceived Quality of Life in Older Adults. *International Journal of Environmental Research and Public Health*, *18*(21), 11515. https://doi.org/10.3390/ijerph182111515

Bedaf, S., Draper, H., Gelderblom, G.-J., Sorell, T., & De Witte, L. (2016). Can a Service Robot Which Supports Independent Living of Older People Disobey a Command? The Views of Older People, Informal Carers and Professional Caregivers on the Acceptability of Robots. *International Journal of Social Robotics*, *8*(3), 409-420. https://doi.org/10.1007/s12369-016-0336-0

Bedaf, S., Marti, P., Amirabdollahian, F., & De Witte, L. (2018). A multi-perspective evaluation of a service robot for seniors : The voice of different stakeholders. *Disability and Rehabilitation: Assistive Technology*, *13*(6), 592-599. https://doi.org/10.1080/17483107.2017.1358300

Beer, J. M., Smarr, C.-A., Chen, T. L., Prakash, A., Mitzner, T. L., Kemp, C. C., & Rogers, W. A. (2012). The domesticated robot : Design guidelines for assisting older adults to age in place. *Proceedings of the Seventh Annual ACM/IEEE International Conference on Human-Robot Interaction*, 335-342. https://doi.org/10.1145/2157689.2157806

Cabrita, M., Tabak, M., & Vollenbroek-Hutten, M. M. (2019). Older Adults' Attitudes Toward Ambulatory Technology to Support Monitoring and Coaching of Healthy Behaviors : Qualitative Study. *JMIR Aging*, *2*(1), e10476. https://doi.org/10.2196/10476

Cao, Y., Erdt, M., Robert, C., Naharudin, N. B., Lee, S. Q., & Theng, Y.-L. (2022). Decision-making Factors Toward the Adoption of Smart Home Sensors by Older Adults in Singapore : Mixed Methods Study. *JMIR Aging*, *5*(2), e34239. https://doi.org/10.2196/34239

Cavallo, F., Esposito, R., Limosani, R., Manzi, A., Bevilacqua, R., Felici, E., Di Nuovo, A., Cangelosi, A., Lattanzio, F., & Dario, P. (2018). Robotic Services Acceptance in Smart Environments With Older Adults : User Satisfaction and Acceptability Study. *Journal of Medical Internet Research*, *20*(9), e264. https://doi.org/10.2196/jmir.9460

Chen, K. (2018). Assistive Technology and Emotions of Older People – Adopting a Positive and Integrated Design Approach. In J. Zhou & G. Salvendy (Éds.), *Human Aspects of IT for the Aged Population. Acceptance, Communication and Participation* (Vol. 10926, p. 21-29). Springer International Publishing. https://doi.org/10.1007/978-3-319-92034-4_2

Chen, K. (2020). Why do older people love and hate assistive technology? – an emotional experience perspective. *Ergonomics*, *63*(12), 1463-1474. https://doi.org/10.1080/00140139.2020.1808714

Chen, T. L., Bhattacharjee, T., Beer, J. M., Ting, L. H., Hackney, M. E., Rogers, W. A., & Kemp, C. C. (2017). Older adults' acceptance of a robot for partner dance-based exercise. *PLOS ONE*, *12*(10), e0182736. https://doi.org/10.1371/journal.pone.0182736

Chiu, C.-J., Lo, Y.-H., Montayre, J., Abu-Odah, H., Chen, M.-L., & Zhao, I. Y. (2023). Identifying Preferred Appearance and Functional Requirements of Aged Care Robots Among Older Chinese Immigrants : Cross-Sectional Study. *JMIR Aging*, *6*, e48646-e48646. https://doi.org/10.2196/48646

Chung, J., Brakey, H. R., Reeder, B., Myers, O., & Demiris, G. (2023). Community-dwelling older adults' acceptance of smartwatches for health and location tracking. *International Journal of Older People Nursing*, *18*(1), e12490. https://doi.org/10.1111/opn.12490

Del Toro García, X., Llumiguano Solano, H., Bolaños Peño, C., Fernández-Bermejo Ruiz, J., Dorado Chaparro, J., Santofimia Romero, M. J., Villanueva Molina, F. J., Sánchez Fábrega, R. A., Díaz Molina, A., Tirado Bejarano, A. J., & Cañuelo Noci, J. (2023). Evaluating the Energy Requirements of Assistive Technologies for Older Adults. In D. Archambault & G. Kouroupetroglou (Éds.), *Studies in Health Technology and Informatics*. IOS Press. https://doi.org/10.3233/SHTI230590

Doering, N., Richter, K., Gross, H.-M., Schroeter, C., Mueller, S., Volkhardt, M., Scheidig, A., & Debes, K. (2015). Robotic Companions for Older People : A Case Study in the Wild. *Studies in Health Technology and Informatics*, *219*, 147-152.

Domingos, C., Costa, P., Santos, N. C., & Pêgo, J. M. (2022). Usability, Acceptability, and Satisfaction of a Wearable Activity Tracker in Older Adults : Observational Study in a Real-Life Context in Northern Portugal. *Journal of Medical Internet Research*, *24*(1), e26652. https://doi.org/10.2196/26652

D'Onofrio, G., Fiorini, L., De Mul, M., Fabbricotti, I., Okabe, Y., Hoshino, H., Limosani, R., Vitanza, A., Greco, F., Giuliani, F., Guiot, D., Senges, E., Kung, A., Cavallo, F., Sancarlo, D., & Greco, A. (2018). Agile Co-Creation for Robots and Aging (ACCRA) Project : New technological solutions for older people. *European Geriatric Medicine*, *9*(6), 795-800. https://doi.org/10.1007/s41999-018-0106-7

Dosso, J. A., Bandari, E., Malhotra, A., Guerra, G. K., Hoey, J., Michaud, F., Prescott, T. J., & Robillard, J. M. (2022). User perspectives on emotionally aligned social robots for older adults and persons living with dementia. *Journal of Rehabilitation and Assistive Technologies Engineering*, *9*, 20556683221108364. https://doi.org/10.1177/20556683221108364

Draper, H., Sorell, T., Bedaf, S., Syrdal, D. S., Gutierrez-Ruiz, C., Duclos, A., & Amirabdollahian, F. (2014). Ethical Dimensions of Human-Robot Interactions in the Care of Older People : Insights from 21 Focus Groups Convened in the UK, France and the Netherlands. In M. Beetz, B. Johnston, & M.-A. Williams (Éds.), *Social Robotics* (Vol. 8755, p. 135-145). Springer International Publishing. https://doi.org/10.1007/978-3-319-11973-1_14

Edwards, K., & Mccluskey, A. (2010). A survey of adult power wheelchair and scooter users. *Disability and Rehabilitation: Assistive Technology*, *5*(6), 411-419. https://doi.org/10.3109/17483101003793412

Eftring, H., & Frennert, S. (2016). Designing a social and assistive robot for seniors. *Zeitschrift Für Gerontologie Und Geriatrie*, *49*(4), 274-281. https://doi.org/10.1007/s00391-016-1064-7

Esmaeilzadeh, P., & Maddah, M. (2024). Robotic companions and healthy aging : A mixed-methods exploration of older adults' perspectives and insights. *Technology in Society*, *78*, 102689. https://doi.org/10.1016/j.techsoc.2024.102689

F. Corbett, C., M. Combs, E., J. Wright, P., L. Owens, O., Stringfellow, I., Nguyen, T., & Van Son, C. R. (2021). Virtual Home Assistant Use and Perceptions of Usefulness by Older Adults and Support Person Dyads. *International Journal of Environmental Research and Public Health*, *18*(3), 1113. https://doi.org/10.3390/ijerph18031113

Fang, T., Cao, W., Chen, C., Zhang, Y., Wang, Z., & Wu, X. (2021). A soft exosuit for hip extension assistance of the elderly. *Technology and Health Care*, *29*(4), 837-841. https://doi.org/10.3233/THC-202423

Farina, N., Sherlock, G., Thomas, S., Lowry, R. G., & Banerjee, S. (2019). Acceptability and feasibility of wearing activity monitors in community-dwelling older adults with dementia. *International Journal of Geriatric Psychiatry*, *34*(4), 617-624. https://doi.org/10.1002/gps.5064

Fiorini, L., Pani, J., Rovini, E., Toccafondi, L., Calamida, N., Lorusso, L., Russo, S., D'Onofrio, G., Iannacone, G., Giuliani, F., Taveter, K., & Cavallo, F. (2024). Promoting Older Adults' Independent Living with Assistive Technology : Emotional Requirements, Technostress and Formal Caregivers Perspective. *2024 46th Annual International Conference of the IEEE Engineering in Medicine and Biology Society (EMBC)*, 1-4. https://doi.org/10.1109/EMBC53108.2024.10782413

Fowe, I. E., & Boot, W. R. (2022). Understanding older adults' attitudes toward mobile and wearable technologies to support health and cognition. *Frontiers in Psychology*, *13*, 1036092. https://doi.org/10.3389/fpsyg.2022.1036092

Gasteiger, N., Ahn, H. S., Gasteiger, C., Lee, C., Lim, J., Fok, C., Macdonald, B. A., Kim, G. H., & Broadbent, E. (2021). Robot-Delivered Cognitive Stimulation Games for Older Adults : Usability and Acceptability Evaluation. *ACM Transactions on Human-Robot Interaction*, *10*(4), 1-18. https://doi.org/10.1145/3451882

Ha, J., & Park, H. K. (2020). Factors Affecting the Acceptability of Technology in Health Care Among Older Korean Adults with Multiple Chronic Conditions : A Cross-Sectional Study Adopting the Senior Technology Acceptance Model. *Clinical Interventions in Aging*, *Volume 15*, 1873-1881. https://doi.org/10.2147/CIA.S268606

Hall, A. K., Backonja, U., Painter, I., Cakmak, M., Sung, M., Lau, T., Thompson, H. J., & Demiris, G. (2019). Acceptance and perceived usefulness of robots to assist with activities of daily living and healthcare tasks. *Assistive Technology*, *31*(3), 133-140. https://doi.org/10.1080/10400435.2017.1396565

Harrington, E. E., Bishop, A. J., Do, H. M., & Sheng, W. (2023). Perceptions of socially assistive robots : A pilot study exploring older adults' concerns. *Current Psychology*, *42*(3), 2145-2156. https://doi.org/10.1007/s12144-021-01627-5

Harris-Gersten, M. L., Davagnino, J. M., Alcorn, E. R., & Hastings, S. N. (2023). Usability and Acceptability of Social Robot Pets Among Community-Dwelling Veterans Living With Dementia and Their Caregivers. *American Journal of Alzheimer's Disease & Other Dementias®*, *38*, 15333175231200973. https://doi.org/10.1177/15333175231200973

Huang, Q., Li, Y., Wu, X., Ge, S., Qu, Z., Wang, A., & Tang, X. (2022). The willingness and influencing factors to choose smart senior care among old adults in China. *BMC Geriatrics*, *22*(1), 967. https://doi.org/10.1186/s12877-022-03691-3

Hudson, J., Ungar, R., Albright, L., Tkatch, R., Schaeffer, J., & Wicker, E. R. (2020). Robotic Pet Use Among Community-Dwelling Older Adults. *The Journals of Gerontology: Series B*, *75*(9), 2018-2028. https://doi.org/10.1093/geronb/gbaa119

Hurmuz, M. Z. M., Jansen-Kosterink, S. M., Mork, P. J., Bach, K., & Hermens, H. J. (2025). Factors influencing the use of an artificial intelligence-based app (SELF BACK) for tailored self-management support among adults with neck and/or low back pain. *Disability and Rehabilitation*, *47*(4), 958-967. https://doi.org/10.1080/09638288.2024.2361811

Ipakchian Askari, S., Huldtgren, A., & IJsselsteijn, W. (2018). Wear It or Fear It—Exploration of Drivers & Barriers in Smartwatch Acceptance by Senior Citizens: *Proceedings of the 4th International Conference on Information and Communication Technologies for Ageing Well and e-Health*, 26-36. https://doi.org/10.5220/0006673000260036

Jafari, N., Lim, M., Hassani, A., Cordeiro, J., Kam, C., & Ho, K. (2022). Human-like tele-health robotics for older adults – A preliminary feasibility trial and vision. *Journal of Rehabilitation and Assistive Technologies Engineering*, *9*, 20556683221140345. https://doi.org/10.1177/20556683221140345

Jaschinski, C., Ben Allouch, S., Peters, O., Cachucho, R., & Van Dijk, J. A. G. M. (2021). Acceptance of Technologies for Aging in Place : A Conceptual Model. *Journal of Medical Internet Research*, *23*(3), e22613. https://doi.org/10.2196/22613

Johnson, A. H., Lee, K., Reeder, B., Popejoy, L., & Vogelsmeier, A. (2025). Feasibility and Acceptability of Smartwatches for Use by Nursing Home Residents. *CIN: Computers, Informatics, Nursing*, *43*(5). https://doi.org/10.1097/CIN.0000000000001245

Jung, S. H., & Shin, Y. S. (2024). Factors associated with intention to use care robots among people with physical disabilities. *Nursing Outlook*, *72*(3), 102145. https://doi.org/10.1016/j.outlook.2024.102145

Kadylak, T., & Cotten, S. R. (2020). United States older adults' willingness to use emerging technologies. *Information, Communication & Society*, *23*(5), 736-750. https://doi.org/10.1080/1369118X.2020.1713848

Kalınkara, V., Başıbüyük, G. Ö., & Ay, F. (2024). The factors affecting acceptance of gerontechnological products by older Turkish adults. *Archives of Gerontology and Geriatrics*, *117*, 105198. https://doi.org/10.1016/j.archger.2023.105198

Kang, H., Yang, T., Banu, N., Ng, S. W. T., & Lee, J. K. (2025). Exploring Smart Health Wearable Adoption Among Singaporean Older Adults Based on Self-Determination Theory : Web-Based Survey Study. *JMIR Aging*, *8*, e69008-e69008. https://doi.org/10.2196/69008

Kaur, A., & Chen, W. (2023). Exploring AI Literacy Among Older Adults. In D. Archambault & G. Kouroupetroglou (Éds.), *Studies in Health Technology and Informatics*. IOS Press. https://doi.org/10.3233/SHTI230589

Kearns, W. D., Becker, A. J., Condon, J. P., Molinari, V., Hanson, A., Conover, W., & Fozard, J. L. (2022). Views of wheelchair users and caregivers regarding a passive safety monitoring system for electric powered wheelchair operators with cognitive impairment. *Assistive Technology*, *34*(1), 64-76. https://doi.org/10.1080/10400435.2019.1689537

Kim, M. Y., Park, J. Y., Leigh, J.-H., Kim, Y. J., Nam, H. S., Seo, H. G., Oh, B.-M., Kim, S., & Bang, M. S. (2022). Exploring user perspectives on a robotic arm with brain–machine interface : A qualitative focus group study. *Medicine*, *101*(36), e30508. https://doi.org/10.1097/MD.0000000000030508

Koceska, N., & Koceski, S. (2022). Supporting elderly's independent living with a mobile robot platform. *JUCS - Journal of Universal Computer Science*, *28*(5), 475-498. https://doi.org/10.3897/jucs.76579

Koceska, N., Koceski, S., Beomonte Zobel, P., Trajkovik, V., & Garcia, N. (2019). A Telemedicine Robot System for Assisted and Independent Living. *Sensors*, *19*(4), 834. https://doi.org/10.3390/s19040834

Koceski, S., & Koceska, N. (2016). Evaluation of an Assistive Telepresence Robot for Elderly Healthcare. *Journal of Medical Systems*, *40*(5), 121. https://doi.org/10.1007/s10916-016-0481-x

Korchut, A., Szklener, S., Abdelnour, C., Tantinya, N., Hernández-Farigola, J., Ribes, J. C., Skrobas, U., Grabowska-Aleksandrowicz, K., Szczęśniak-Stańczyk, D., & Rejdak, K. (2017). Challenges for Service Robots—Requirements of Elderly Adults with Cognitive Impairments. *Frontiers in Neurology*, *8*, 228. https://doi.org/10.3389/fneur.2017.00228

Le Deist, F., & Latouille, M. (2016). Acceptability Conditions for Telemonitoring Gerontechnology in the Elderly. *IRBM*, *37*(5-6), 284-288. https://doi.org/10.1016/j.irbm.2015.12.002

Lei, J., Liang, Y., Su, Z., Dong, P., Liang, J., & Lin, L. (2024). Can Socially Assistive Robots Be Accepted by Older People Living Alone in the Community? : Empirical Findings from a Social Work Project in China. *Journal of Gerontological Social Work*, *67*(7), 995-1012. https://doi.org/10.1080/01634372.2024.2339992

Lesauskaitė, V., Damulevičienė, G., Knašienė, J., Kazanavičius, E., Liutkevičius, A., & Janavičiūtė, A. (2019). Older Adults—Potential Users of Technologies. *Medicina*, *55*(6), 253. https://doi.org/10.3390/medicina55060253

Leung, A. (s. d.). *The Presence of Humanoid Robot With Older Adults at Homes | Cochrane Library*. https://doi.org/10.1002/central/CN-02729455

Li, M., & Woolrych, R. (2021). Experiences of Older People and Social Inclusion in Relation to Smart "Age-Friendly" Cities : A Case Study of Chongqing, China. *Frontiers in Public Health*, *9*, 779913. https://doi.org/10.3389/fpubh.2021.779913

Lin, X. Y., Boot, W., Charness, N., Czaja, S. J., & Rogers, W. (2025). Characterizing the attitudes, proficiency, and usage patterns for daily technology among older adults with a cognitive impairment. *Disability and Rehabilitation: Assistive Technology*, *20*(6), 1677-1686. https://doi.org/10.1080/17483107.2025.2460104

Lin, X. Y., Moxley, J., Sharit, J., & Czaja, S. J. (2025). Beyond the Digital Divide : Factors Associated With Adoption of Technologies Related to Aging in Place. *Journal of Applied Gerontology*, *44*(6), 959-969. https://doi.org/10.1177/07334648251318789

Martin-Hammond, A., Vemireddy, S., & Rao, K. (2019). Exploring Older Adults' Beliefs About the Use of Intelligent Assistants for Consumer Health Information Management : A Participatory Design Study. *JMIR Aging*, *2*(2), e15381. https://doi.org/10.2196/15381

Mitseva, A., Peterson, C. B., Karamberi, C., Oikonomou, L. Ch., Ballis, A. V., Giannakakos, C., & Dafoulas, G. E. (2012). Gerontechnology : Providing a Helping Hand When Caring for Cognitively Impaired Older Adults—Intermediate Results from a Controlled Study on the Satisfaction and Acceptance of Informal Caregivers. *Current Gerontology and Geriatrics Research*, *2012*, 1-19. https://doi.org/10.1155/2012/401705

Möller, J., Stara, V., Amabili, G., Barbarossa, F., Riccardi, G. R., Martella, C., Di Donna, V., Palmier, C., Ogawa, T., Luc, M., Wieching, R., Maranesi, E., & Bevilacqua, R. (2024). Toward Innovation in Healthcare : An Analysis of the Digital Behavior of Older People in Europe and Japan for the Introduction of a Technological Coaching System. *Healthcare*, *12*(2), 143. https://doi.org/10.3390/healthcare12020143

Monden, K. R., Charlifue, S., Philippus, A., Kilbane, M., Muston-Firsch, E., MacIntyre, B., Welch, A., Baldessari, J., Coker, J., & Morse, L. R. (2024). Exploring perspectives on assistive technology use : Barriers, facilitators, and access. *Disability and Rehabilitation: Assistive Technology*, *19*(4), 1676-1686. https://doi.org/10.1080/17483107.2023.2227235

Murciano-Hueso, A., Martín-Lucas, J., Serrate González, S., & Torrijos Fincias, P. (2022). Use and perception of gerontechnology : Differences in a group of Spanish older adults. *Quality in Ageing and Older Adults*, *23*(3), 114-128. https://doi.org/10.1108/QAOA-02-2022-0010

Olatunji, S. A., Bily, M. P., Bayles, M. A., & Rogers, W. A. (2025). Perspectives of Older Adults on Assistive Robot Support at Home. *2025 International Conference On Rehabilitation Robotics (ICORR)*, 1009-1014. https://doi.org/10.1109/ICORR66766.2025.11063150

Orellano-Colón, E., Jutai, J., Santiago, A., Torres, V., Benítez, K., & Torres, M. (2016). Assistive Technology Needs and Measurement of the Psychosocial Impact of Assistive Technologies for Independent Living of Older Hispanics : Lessons Learned. *Technologies*, *4*(3), 21. https://doi.org/10.3390/technologies4030021

Orellano-Colon, E. M., Fernández-Torres, A., Figueroa-Alvira, N., Ortiz-Vélez, B., Rivera-Rivera, N. L., Torres-Ferrer, G. A., & Martín-Payo, R. (2024). Empowering Potential of the My Assistive Technology Guide : Exploring Experiences and User Perspectives. *Disabilities*, *4*(2), 303-320. https://doi.org/10.3390/disabilities4020020

Orellano-Colón, E. M., Rivero-Méndez, M., Lizama, M., & Jutai, J. W. (2018). Assistive technology unmet needs of independent living older Hispanics with functional limitations. *Disability and Rehabilitation: Assistive Technology*, *13*(2), 194-200. https://doi.org/10.1080/17483107.2017.1300693

Peek, S. T. M., Luijkx, K. G., Rijnaard, M. D., Nieboer, M. E., Van Der Voort, C. S., Aarts, S., Van Hoof, J., Vrijhoef, H. J. M., & Wouters, E. J. M. (2016). Older Adults' Reasons for Using Technology while Aging in Place. *Gerontology*, *62*(2), 226-237. https://doi.org/10.1159/000430949

Penteridis, L., D'Onofrio, G., Sancarlo, D., Giuliani, F., Ricciardi, F., Cavallo, F., Greco, A., Trochidis, I., & Gkiokas, A. (2017). Robotic and Sensor Technologies for Mobility in Older People. *Rejuvenation Research*, *20*(5), 401-410. https://doi.org/10.1089/rej.2017.1965

Peters, J., Anvari, B., Licher, J., Wiese, M., Raatz, A., & Wurdemann, H. A. (2025). Acceptance and Usability of a Soft Robotic, Haptic Feedback Seat for Autonomy Level Transitions in Highly Automated Vehicles. *IEEE Transactions on Haptics*, *18*(1), 58-72. https://doi.org/10.1109/TOH.2024.3392473

Pettersson, C., Iwarsson, S., & Månsson Lexell, E. (2015). Experiences of using powered wheelchair or powered scooter and accessibility in housings. *Studies in Health Technology and Informatics*, *217*, 1017-1023.

Pigini Lucia, Facal David, Blasi Lorenzo, & Andrich Renzo. (2011). Tele-Operated Robots in Elderly Care at Home : A Survey on Needs and Perceptions of Elderly People and Caregivers. In *Assistive Technology Research Series*. IOS Press. https://doi.org/10.3233/978-1-60750-814-4-542

Pilissy, T., Toth, A., Fazekas, G., Sobjak, A., Rosenthal, R., Luftenegger, T., Panek, P., & Mayer, P. (2017). Towards a situation-and-user-aware multi-modal motorized toilet system to assist older adults with disabilities : A user requirements study. *2017 International Conference on Rehabilitation Robotics (ICORR)*, 959-964. https://doi.org/10.1109/ICORR.2017.8009373

Pino, M., Dacunha, S., Berger, É., GOnÇALVES, A., & Rigaud, A.-S. (2021). Intérêt de la robotique sociale et d'assistance auprès des sujets âgés. *Actualités Pharmaceutiques*, *60*(611), 36-39. https://doi.org/10.1016/j.actpha.2021.10.010

Pripfl, J., Körtner, T., Batko-Klein, D., Hebesberger, D., Weninger, M., & Gisinger, C. (2016). Social service robots to support independent living : Experiences from a field trial. *Zeitschrift Für Gerontologie Und Geriatrie*, *49*(4), 282-287. https://doi.org/10.1007/s00391-016-1067-4

Randall, K. N., Drew, H., Gilman, E. S., & Dixon, E. (2025). Assistive Technology Uses and Barriers in the Home and Workplace for Adults With Intellectual and Developmental Disabilities. *Journal of Applied Research in Intellectual Disabilities*, *38*(1), e13306. https://doi.org/10.1111/jar.13306

Rantanen, T., Lehto, P., Vuorinen, P., & Coco, K. (2018). Attitudes towards care robots among Finnish home care personnel – a comparison of two approaches. *Scandinavian Journal of Caring Sciences*, *32*(2), 772-782. https://doi.org/10.1111/scs.12508

Sadler, J. R., Khan, A., Lwin, M., & Mubin, O. (2024). Social Robots for Meaningful Seated Activities : Acceptance & Use by Older Adults. *Healthcare*, *12*(13), 1334. https://doi.org/10.3390/healthcare12131334

Sánchez, V. G., Anker-Hansen, C., Taylor, I., & Eilertsen, G. (2019). Older People's Attitudes And Perspectives Of Welfare Technology In Norway. *Journal of Multidisciplinary Healthcare*, *Volume 12*, 841-853. https://doi.org/10.2147/JMDH.S219458

Santini, S., Galassi, F., Kropf, J., & Stara, V. (2020). A Digital Coach Promoting Healthy Aging among Older Adults in Transition to Retirement : Results from a Qualitative Study in Italy. *Sustainability*, *12*(18), 7400. https://doi.org/10.3390/su12187400

Sathananthan, S., Blackburn, É., Kairy, D., Gagnon, D. H., Miller, W. C., Musselman, K., Routhier, F., & Archambault, P. S. (2025). Exploring the challenges in manual wheelchair operation for new users. *Disability and Rehabilitation: Assistive Technology*, *20*(5), 1385-1394. https://doi.org/10.1080/17483107.2024.2448724

Schlomann, A., Seifert, A., Zank, S., & Rietz, C. (2020). Assistive Technology and Mobile ICT Usage Among Oldest-Old Cohorts : Comparison of the Oldest-Old in Private Homes and in Long-Term Care Facilities. *Research on Aging*, *42*(5-6), 163-173. https://doi.org/10.1177/0164027520911286

Seelye, A. M., Wild, K. V., Larimer, N., Maxwell, S., Kearns, P., & Kaye, J. A. (2012). Reactions to a Remote-Controlled Video-Communication Robot in Seniors' Homes : A Pilot Study of Feasibility and Acceptance. *Telemedicine and E-Health*, *18*(10), 755-759. https://doi.org/10.1089/tmj.2012.0026

Shareef, M. A., Kumar, V., Dwivedi, Y. K., Kumar, U., Akram, M. S., & Raman, R. (2021). A new health care system enabled by machine intelligence : Elderly people's trust or losing self control. *Technological Forecasting and Social Change*, *162*, 120334. https://doi.org/10.1016/j.techfore.2020.120334

Silva, L. M., Souza, A. C. D., Fhon, J. R. S., Rodrigues, R. A. P., Santos, L. A. D., & Gomes, M. F. C. T. (2022). Factors associated with the use of assistive technologies in elders in their home environments. *Rev Rene*, *23*, e78534. https://doi.org/10.15253/2175-6783.20222378534

Silvius, H. A. M., Tak, E. C. P. M., Mook-Kanamori, D. O., Vos, H. M. M., Numans, M. E., & Chavannes, N. H. (2020). Effects of Technology Use on Ageing in Place : The iZi Pilots. *International Journal of Environmental Research and Public Health*, *17*(14), 5052. https://doi.org/10.3390/ijerph17145052

Smarr, C.-A., Mitzner, T. L., Beer, J. M., Prakash, A., Chen, T. L., Kemp, C. C., & Rogers, W. A. (2014). Domestic Robots for Older Adults : Attitudes, Preferences, and Potential. *International Journal of Social Robotics*, *6*(2), 229-247. https://doi.org/10.1007/s12369-013-0220-0

Smarr, C.-A., Prakash, A., Beer, J. M., Mitzner, T. L., Kemp, C. C., & Rogers, W. A. (2012). Older Adults' Preferences for and Acceptance of Robot Assistance for Everyday Living Tasks. *Proceedings of the Human Factors and Ergonomics Society Annual Meeting*, *56*(1), 153-157. https://doi.org/10.1177/1071181312561009

Timon, C. M., Heffernan, E., Kilcullen, S., Hopper, L., Lee, H., Gallagher, P., Smeaton, A. F., Moran, K., Hussey, P., & Murphy, C. (2024). Developing Independent Living Support for Older Adults Using Internet of Things and AI-Based Systems : Co-Design Study. *JMIR Aging*, *7*, e54210-e54210. https://doi.org/10.2196/54210

Tobis, S., Piasek, J., Cylkowska-Nowak, M., & Suwalska, A. (2022). Robots in Eldercare : How Does a Real-World Interaction with the Machine Influence the Perceptions of Older People? *Sensors*, *22*(5), 1717. https://doi.org/10.3390/s22051717

Tsai, H. S., Shillair, R., Cotten, S. R., Winstead, V., & Yost, E. (2015). Getting Grandma Online : Are Tablets the Answer for Increasing Digital Inclusion for Older Adults in the U.S.? *Educational Gerontology*, *41*(10), 695-709. https://doi.org/10.1080/03601277.2015.1048165

Van Assche, M., Petrovic, M., Cambier, D., Calders, P., Van Gelder, P., Werner, F., & Van De Velde, D. (2024). Socially Assistive Robots in Aged Care : Expectations of Older Adults with MCI in Assisted Living Facilities and Their Caregivers. *International Journal of Social Robotics*, *16*(4), 687-698. https://doi.org/10.1007/s12369-024-01115-3

Vaportzis, E., Giatsi Clausen, M., & Gow, A. J. (2017). Older Adults Perceptions of Technology and Barriers to Interacting with Tablet Computers : A Focus Group Study. *Frontiers in Psychology*, *8*, 1687. https://doi.org/10.3389/fpsyg.2017.01687

Verloo, H., Kampel, T., Vidal, N., & Pereira, F. (2020). Perceptions About Technologies That Help Community-Dwelling Older Adults Remain at Home : Qualitative Study. *Journal of Medical Internet Research*, *22*(6), e17930. https://doi.org/10.2196/17930

Wang, R. H., Sudhama, A., Begum, M., Huq, R., & Mihailidis, A. (2017). Robots to assist daily activities : Views of older adults with Alzheimer's disease and their caregivers. *International Psychogeriatrics*, *29*(1), 67-79. https://doi.org/10.1017/S1041610216001435

Wang, S., Bolling, K., Mao, W., Reichstadt, J., Jeste, D., Kim, H.-C., & Nebeker, C. (2019). Technology to Support Aging in Place : Older Adults' Perspectives. *Healthcare*, *7*(2), 60. https://doi.org/10.3390/healthcare7020060

Wang, Y., Sani, N. Md., Hua, Y., Jiang, Q., & Zhao, L. (2024). Factors affecting the elderly's behavioral intention toward smart home systems : A cross-sectional study from China's eastern coast. *PLOS ONE*, *19*(12), e0311280. https://doi.org/10.1371/journal.pone.0311280

Werner, C., Dometios, A. C., Tzafestas, C. S., Maragos, P., Bauer, J. M., & Hauer, K. (2022). Evaluating the task effectiveness and user satisfaction with different operation modes of an assistive bathing robot in older adults. *Assistive Technology*, *34*(2), 222-231. https://doi.org/10.1080/10400435.2020.1755744

Werner, C., Geravand, M., Korondi, P. Z., Peer, A., Bauer, J. M., & Hauer, K. (2020). Evaluating the sit-to-stand transfer assistance from a smart walker in older adults with motor impairments. *Geriatrics & Gerontology International*, *20*(4), 312-316. https://doi.org/10.1111/ggi.13874

Wichmann, L. J., Reiff, N., Zintl, S., & Berking, M. (2021). Developing a digital assistive tool for dementia patients and their caregivers : A qualitative study on needs, potential functions, practical requirements, user acceptance, and ethical considerations. *Alzheimer's & Dementia*, *17*(S11), e056015. https://doi.org/10.1002/alz.056015

Wiegel, P., Fotteler, M. L., Kohn, B., Mayer, S., Verri, F. M., Dallmeier, D., & Denkinger, M. (2024). Perceived Benefit and Satisfaction With a Tablet Computer and an Emergency Smartwatch by Older Adults and Their Relatives : Prospective Real-World Pilot Study. *JMIR Human Factors*, *11*, e53811-e53811. https://doi.org/10.2196/53811

Wilkowska, W., Offermann, J., Spinsante, S., Poli, A., & Ziefle, M. (2022). Analyzing technology acceptance and perception of privacy in ambient assisted living for using sensor-based technologies. *PLOS ONE*, *17*(7), e0269642. https://doi.org/10.1371/journal.pone.0269642

Wong, A. K. C., Bayuo, J., Su, J. J., Chow, K. K. S., Wong, S. M., Wong, B. P., Lee, A. Y. L., & Wong, F. K. Y. (2024). Exploring the Experiences of Community-Dwelling Older Adults on Using Wearable Monitoring Devices With Regular Support From Community Health Workers, Nurses, and Social Workers : Qualitative Descriptive Study. *Journal of Medical Internet Research*, *26*, e49403. https://doi.org/10.2196/49403

Zhang, J., Wang, H., Li, Q., & Luximon, Y. (2024). What Is the Real-Life Experience of Older Adults on Smart Healthcare Technologies? An Exploratory Interview Study. *Gerontology*, *70*(9), 978-990. https://doi.org/10.1159/000539539

Zheng, L., Foley, K.-R., Grove, R., Elley, K., Brown, S. A., Leong, D., Li, X., Pellicano, E., Trollor, J. N., & Hwang, Y. I. (Jane). (2022). The use of everyday and assistive technology in the lives of older autistic adults. *Autism*, *26*(6), 1550-1562. https://doi.org/10.1177/13623613211058519
