# Supplementary material for: Acceptance of AI-based assistive technologies for autonomy support among older adults: a scoping review
Source: J Gerontol A Biol Sci Med Sci. 2026 Sep 21;81(11):glag238. doi: 10.1093/gerona/glag238 (PMC13718428; doi:10.1093/gerona/glag238)
Supplement: Supplementary file 1 [file glag238_supplementary_data.zip › Appendix 1.pdf]

Acceptance of AI-Based Assistive Technologies for Autonomy Support Among Older Adults: A Scoping Review

Pubmed

(((((((((((((((((((aged[MeSH Terms]) OR (aging[MeSH Terms])) OR (cognitive aging[MeSH Terms])) OR (frail elderly[MeSH Terms])) OR (aged[Title/Abstract])) OR (aging[Title/Abstract])) OR (cognitive aging[Title/Abstract])) OR (frail elderly[Title/Abstract])) OR (elderly[Title/Abstract])) OR (elder[Title/Abstract])) OR (frail[Title/Abstract])) OR (frail elder[Title/Abstract])) OR (ageing people[Title/Abstract])) OR (older adults[Title/Abstract])) OR (Adult[Title/Abstract])) OR (Frail Older[Title/Abstract])) OR (frail older adults[Title/Abstract])) OR (senior[Title/Abstract])) AND (((intelligent systems[MeSH Terms]) OR (artificial intelligence[MeSH Terms])) OR (generative artificial intelligence[MeSH Terms])) OR (voice recognition[MeSH Terms])) OR (robotics[MeSH Terms])) OR (self-help devices[MeSH Terms])) OR (machine learning[MeSH Terms])) OR (equipment and supplies[MeSH Terms])) OR (intelligent systems[Title/Abstract])) OR (artificial intelligence[Title/Abstract])) OR (generative artificial intelligence[Title/Abstract])) OR (voice recognition[Title/Abstract])) OR (robotics[Title/Abstract])) OR (self-help devices[Title/Abstract])) OR (machine learning[Title/Abstract])) OR (equipment[Title/Abstract] AND supplies[Title/Abstract])) OR (intelligence computational[Title/Abstract])) OR (intelligence computer[Title/Abstract])) OR (generative AI[Title/Abstract])) OR (new technology[Title/Abstract])) OR (digital innovation[Title/Abstract])) OR (automatic device[Title/Abstract])) OR (apparatus[Title/Abstract] AND instruments[Title/Abstract])) OR (supplies[Title/Abstract] AND equipment[Title/Abstract])) OR (supplies[Title/Abstract])) OR (equipment[Title/Abstract])) OR (medical device[Title/Abstract])) OR (machine[Title/Abstract])) OR (learning[Title/Abstract])) OR (Assistive technology[Title/Abstract])) OR (robotic technologies[Title/Abstract])) OR (gerontechnology[Title/Abstract])) AND ((((((((((((((((((((((((personal autonomy[MeSH Terms]) OR (independent living[MeSH Terms])) OR (activities of daily living[MeSH Terms])) OR (healthy aging[MeSH Terms])) OR (relational autonomy[MeSH Terms])) OR (mobility limitation[MeSH Terms])) OR (aging in place[MeSH Terms])) OR (functional status[MeSH Terms])) OR (personal autonomy[Title/Abstract])) OR (independent living[Title/Abstract])) OR (activities of daily living[Title/Abstract])) OR (healthy aging[Title/Abstract])) OR (mobility limitation[Title/Abstract])) OR (aging in place[Title/Abstract])) OR (functional status[Title/Abstract])) OR (autonomy[Title/Abstract])) OR (free will[Title/Abstract])) OR (self determination[Title/Abstract])) OR (community dwelling[Title/Abstract])) OR (ageing well[Title/Abstract])) OR (relational autonomy[Title/Abstract])) OR (independent lifestyle[Title/Abstract])) OR (self-sufficiency[Title/Abstract]))) AND (((((((((((((((((((((((((((((((((((((adoption[MeSH Terms]) OR (health services accessibility[MeSH Terms])) OR (behavior[MeSH Terms])) OR (patient compliance[MeSH Terms])) OR (compliance[MeSH Terms])) OR (attitude[MeSH Terms])) OR (Health Equity[MeSH Terms])) OR (adoption[Title/Abstract])) OR (health services accessibility[Title/Abstract])) OR (behavior[Title/Abstract])) OR (patient compliance[Title/Abstract])) OR (compliance[Title/Abstract])) OR (attitude[Title/Abstract])) OR (Health Equity[Title/Abstract])) OR (Opinion[Title/Abstract])) OR (use[Title/Abstract])) OR (utilization[Title/Abstract])) OR (usage[Title/Abstract])) OR (Access to Care[Title/Abstract])) OR (Accessibilities[Title/Abstract])) OR (Equity in Health[Title/Abstract])) OR (Health Services[Title/Abstract])) OR (availability of health services[Title/Abstract])) OR (Adherence[Title/Abstract])) OR (cooperation[Title/Abstract])) OR (Sentiments[Title/Abstract])) OR (Equity[Title/Abstract])) OR (Acceptance[Title/Abstract])) OR (acceptability[Title/Abstract])) OR (Accessibility[Title/Abstract])) OR (perceived usefulness[Title/Abstract])) OR (Digital Divide[Title/Abstract])) OR (Practice[Title/Abstract])) OR (barriers[Title/Abstract])) OR (facilitators[Title/Abstract])) OR (levers[Title/Abstract]))) AND (("2010/01/01"[Date - Publication] : "3000"[Date - Publication]))) NOT (Systematic Review[Publication Type])) NOT (Review[Publication Type])) NOT (Editorial[Publication Type])) NOT (Letter[Publication Type])) NOT (Comment[Publication Type])) NOT (Case Reports[Publication Type])) NOT (Practice Guideline[Publication Type])) NOT (Consensus Development Conference[Publication Type])) NOT (Historical Article[Publication Type]))
